# Synthesis and Polymerization of a Lactone Building Block Derived From Cashew Nut Shells

**DOI:** 10.1002/cssc.71014

**Published:** 2026-10-12

**Authors:** Paulo R. Wohlfahrt, Martina Huber, Kerstin Elsinghorst, Seraina Mihailescu, Hannah Cumming, Stefan Naumann, Harald Gröger

**Affiliations:** ^1^ Chair of Industrial Organic Chemistry and Biotechnology Faculty of Chemistry Bielefeld University Bielefeld Germany; ^2^ Institute for Macromolecular Chemistry Albert‐Ludwigs‐University of Freiburg Freiburg Germany; ^3^ Freiburg Materials Research Center (FMF) Albert‐Ludwigs‐University of Freiburg Freiburg Germany

**Keywords:** Baeyer–Villiger oxidation, caprolactone, cardanol, copolymer, crystallinity, monomer, poly(*ε*‐caprolactone), polymer, polymer chemistry, polymerization

## Abstract

The biorenewable feedstock cardanol, a phenol compound substituted with an unsaturated C15 hydrocarbon chain, was selectively transformed into a cyclohexanone derivative through partial hydrogenation. Subsequent conversion via Baeyer–Villiger oxidation (BVO) gave a lactone product (pentadecylcaprolactone, PDCL), which is a mixture of two regioisomers. For the BVO, two variants that are based on either the direct use of a perbenzoic acid as reagent or alternatively the use of a readily available aldehyde in the presence of molecular oxygen as an attractive oxidation reagent have been studied and turned out to be feasible. In addition, suitable work‐up has been demonstrated that furnished a sample of the cardanol‐derived PDCL‐type lactone in 84% isolated yield with 99% purity. Using metal‐free polymerization techniques, a PDCL sample has then been readily converted into its homopolymer, which displays a low crystallinity, the latter due to moderate side‐chain crystallization. Importantly, in a statistical copolymer with *ε*‐caprolactone (CL), PDCL was shown to be a useful agent to modulate crystallinity, resulting in copolyesters with reduced or very minor crystallinity and accordingly low melting points. For such an effect, typically expensive and fossil‐based monomers such as δ‐valerolactone (VL) have to be used, highlighting the potential of PDCL for practical application.

## Graphical abstract

Cardanol as a renewable substituted phenol feedstock was selectively transformed into the corresponding cyclohexanone derivative by means of a partial metal‐catalyzed hydrogenation. Subsequent conversion via Baeyer–Villiger oxidation gave a lactone product consisting of a mixture of regioisomers, which has been readily converted into its homopolymer displaying a low crystallinity. In addition, a statistical copolymer with ε‐caprolactone was prepared.

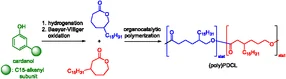

## 1 Introduction

Fats and oils derived from natural plants are a large resource stream for the chemical industry. Next to products which come from sunflower, olive, rapeseed, and flax sources, waste streams from the treatment of natural resource streams can also serve as potential feedstocks [1].

Vegetable oils are commonly used as feedstocks for producing oleochemicals, largely due to the well‐established chemistry of their alkene groups. Typical reactions include epoxidation, hydroxylation, hydroformylation, alkoxycarbonylation, oxidation, hydrogenation, and metathesis of unsaturated fatty acids [2]. For instance, the Wacker oxidation of methyl oleate has been reported as a valuable transformation [3].

Besides vegetable oils, waste products from cashew nut processing offer another important feedstock. The cashew fruit consists of a sweet cashew apple and the cashew nut enclosed in its shell. While the apple and nut are the primary products, the cashew nut shell is generally a byproduct [4]. This shell contains an oily substance known as cashew nut shell liquid (CNSL), which is extracted mainly by solvent or hot extraction methods, with an annual production exceeding one million tons [5, 6]. Alternative extraction techniques, such as using hot vegetable oil, have been developed to reduce the environmental impact and carbon footprint of the process [7].

The majority of CNSL production takes place in the countries Brazil, India, Indonesia, Vietnam, and Thailand [8]. The CNSL primarily contains four main components: anacardic acid, cardanol, cardol, and methylcardol (Figure 1). Each of these compounds features a C15 aliphatic side chain with varying degrees of unsaturation. The typical composition of these constituents is also illustrated in Figure 1 [4].

**FIGURE 1:**
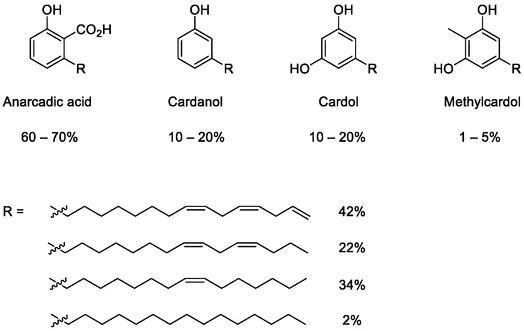
Composition of common CNSL by mass [4].

Thermal decarboxylation of the CNSL converts the main component anacardic acid to cardanol, and after distillation pure cardanol is obtained in isolated form [9].

Cardanol is highly valued in industry for its environmentally friendly profile, chemical adaptability, and its ability to serve as a renewable alternative to fossil‐derived materials [10]. In phenolic resin production, cardanol can replace up to 30% of phenol, with plywood manufacturing being a major application [11]. It is also a key ingredient in the synthesis of curing agents for robust epoxy coatings, which are widely used on concrete floors, in marine settings, and on various industrial surfaces. Many of these curing agents are phenalkamines, known for their strong adhesive properties, particularly in metal‐to‐metal bonding [12].

Due to its excellent compatibility with other organic compounds, cardanol is commonly used as an additive in paints and coatings. Its derivatives, such as ethoxylated and sulfonated cardanol, are utilized as emulsifiers in pesticides and as wetting agents in the textile industry [4]. Phosphorylation enables the production of cardanol‐based materials with excellent flame‐retardant properties [13]. Beyond this, cardanol can be used to create advanced materials, such as antimicrobial paints incorporating silver nanoparticles [14].

Cardanol's chemical structure allows for a variety of transformations. Similar to fatty acids, its unsaturated side chain can be epoxidized to yield valuable intermediates. The phenolic moiety can also undergo modifications like etherification and acylation, enabling the synthesis of numerous monomers, including cardanol benzoxazines [15].

In our research described in the following, we have developed a straightforward and potentially scalable synthesis of the cardanol‐derived lactone pentadecylcaprolactone (PDCL) and explored its copolymerization with *ε*‐caprolactone (CL). While the synthesis of PDCL has been reported in literature [16, 17], a potentially industrially viable synthesis route has not yet been established. Furthermore, polymerization of this monomer has previously only been described in a single case, and in this instance, a metal‐based catalyst has been employed (SnOct_2_) [16]. Metal‐free polymerization, which aligns especially well with the sustainability focus of this work, has not been reported for PDCL to date.

## 2 Materials and Methods

### 2.1 General

All chemicals and solvents used in this study were sourced from Carl Roth, Fisher Scientific, Merck KGaA, Sigma‐Aldrich, TCI, BLDpharm, and VWR. They were used in analytical or HPLC grade without further purification. Cardanol was obtained from cardolite as NX‐2026 Multipurpose Resin Modifier. Meta‐chloroperbenzoic acid (*m*CPBA) was used with a purity of 75%, the rest being water as a stabilizer. The palladium catalyst was sourced from Sigma‐Aldrich with a loading of 10 wt% palladium. Analysis of the product mixtures was carried out using a 500 MHz NMR spectrometer (Bruker Advance 500).

Analysis of the (co)polymer samples in terms of relative molar masses (*M*_*n*_) and molar mass distribution (*Ð*_M_) was completed using an Agilent Technologies 1260 Series instrument for gel permeation chromatography (GPC) against polystyrene standards in CHCl_3_. A sample concentration of 2 mg/mL, an injection volume of 100 µL, and a flow rate of 1.0 mL min^−1^ was applied when carrying out the measurements. NMR spectra of polymeric samples were recorded in CDCl_3_ on a Bruker Avance Neo 400 (^1^H NMR at 400 MHz and ^13^C NMR at 101 MHz). Thermogravimetric analysis (TGA) was conducted on a Netzsch STA 449 F5. Thermograms were recorded under an oxygen atmosphere using a heating rate of 10 K min^−1^, from 30 to 600 °C. Differential scanning calorimetry (DSC) was measured on a Netzsch DSC 204 F1. Thermograms were recorded using a heating rate of 10 K min^−1^, from −100 to 200 °C.

### 2.2 Selective Hydrogenation of Cardanol

#### 2.2.1 Safety Information

Working with hydrogen gas is extremely dangerous due to its unique physical and chemical properties. Hydrogen is highly flammable and forms explosive mixtures with air, even in small concentrations. Even minor equipment failures or seal leaks can lead to rapid gas release, posing fire, explosion, and asphyxiation risks. Proper ventilation, leak detection systems, and the use of certified‐rated equipment are essential to ensure safety.

#### 2.2.2 Procedure for Hydrogenation of Cardanol (Selected Example)

An autoclave was charged with cardanol (10 g, 33.5 mmol, 1 eq.) and Pd/C (140 mg, 0.13 mmol, 0.4 mol%). The vessel was sealed and sequentially purged with nitrogen and hydrogen. Hydrogen pressure was then adjusted to 60 bar, and the reaction was carried out under controlled conditions (100 °C, 20 h, and 1000 rpm).

After cooling to room temperature, the heterogeneous catalyst was removed by syringe filtration (0.45 µm). The composition of the product mixture was determined by ^1^H NMR spectroscopy.

Purification of the resulting ketone was achieved by dissolving the crude product in cyclohexane (approximately 250 g/L) and adding triethylamine (100 wt%). The solution was filtered through a column of basic alumina, after which the solvent and residual triethylamine were removed by vacuum distillation. This procedure afforded the ketone in 85% yield and with 99% purity.

### 2.3 Baeyer–Villiger Oxidation with *m*CPBA

The substituted ketone (10 g, 32.4 mmol, 1 eq.), which was obtained from hydrogenation of cardanol, was dissolved in dichloromethane (40 mL). *m*CPBA (75%, 11.2 g, 48.6 mmol, 1.5 eq.) was added portionwise over 30 min. The reaction mixture was allowed to react for over 90 min at room temperature. Any leftover peracid and formed carboxylic acid were washed out with NaOH solution (10%, 3 x 30 mL) and brine (1 x 30 mL), and the organic phase was dried over MgSO_4_. After filtering off the drying agent and removing the solvent in vacuo, the lactones were obtained in a yield of 84% as a colorless solid.

### 2.4 Baeyer–Villiger Oxidation under Mukaiyama Conditions

#### 2.4.1 Safety Information

Working with oxygen gas presents serious hazards because oxygen greatly accelerates combustion. Materials that are normally nonflammable can ignite easily or burn explosively in pure oxygen environments. Even small leaks near oils, greases, or organic materials can lead to violent fires or explosions. Strict cleanliness, compatible materials, and proper ventilation are essential precautions when handling pure oxygen gas.

#### 2.4.2 Procedure for Baeyer‐Villiger Oxidation under Mukaiyama Conditions (Selected Example)

The substituted cyclohexanone (154 mg, 0.5 mmol, 1 eq.) and benzaldehyde (212 mg, 2 mmol, 4 eq.) were dissolved in 1,2‐dichloroethane (4 mL) in a 10 mL glass vial. The vessel was flushed with oxygen and then sealed to maintain an oxygen atmosphere. The reaction mixture was stirred at 500 rpm for 24 h at 50 °C. Product composition was determined by ^1^H NMR spectroscopic analysis.

### 2.5 Structural Analysis of Lactones

The lactones (100 mg, 0.3 mmol, 1 eq.), tin(II) 2‐ethylhexanoate (12.5 mg, 30 μmol, 10 mol%), and benzyl alcohol (333 mg, 3.1 mmol, 10 eq.) were combined in a 10 mL glass vial and heated to 130 °C. The reaction mixture was stirred for 2 h. Product composition was analyzed by ^1^H NMR spectroscopy.

### 2.6 Homopolymerization

A stock solution of 1,5,7‐triazabicyclodec‐5‐ene (TBD) and benzyl alcohol in dichloromethane (DCM) was prepared. 100 µL of the stock solution containing TBD (2.2 mg, 16 µmol, 5 eq.) and benzyl alcohol (0.7 mg, 6 µmol, 2 eq.) were added to a 5 mL pressure tube under an inert nitrogen atmosphere. The solvent was evaporated and PDCL (200 mg, 0.6 mmol, 200 eq.) was added. The reaction mixture was stirred at 60 °C for 4 h, cooled down, resolved in DCM, and precipitated in cold methanol.

Conversion of the polymerization was calculated using ^1^H NMR spectroscopy in CDCl_3_ by comparison of the area of the PDCL monomer (2.6–2.5 ppm) and poly(PDCL) (2.3–2.2 ppm) corresponding signal. To obtain the molar mass and dispersity of the resulting polymer, GPC was conducted in CHCl_3_ as the solvent. Thermal properties were analyzed using DSC and TGA.

### 2.7 Copolymerization

All copolymerization reactions were carried out under an inert nitrogen atmosphere in a glovebox. PDCL (130 mg, 0.402 mmol, 50 eq.),  CL (42.62 μL, 0.40 mmol, 50 eq.), the catalytic system (TBD (1.4 mg, 0.010 mmol, 1.25 eq.), 1‐[3,5‐bis(trifluoromethyl)phenyl]‐3‐cyclohexylthiourea (TU, 4.2 mg, 0.010 mmol, 1.25 eq.)), and the initiator BnOH (1.04 μL, 0.010 mmol, 1.25 eq.) were combined and stirred at 60 °C for 1 h. The sample was removed from the glovebox, cooled down, resolved in DCM, and precipitated from cold methanol.

Conversion was calculated analogously to homopolymerization by comparison of monomer and polymer signals, respectively. To obtain the molar mass and dispersity of the resulting polymer, GPC was conducted in CHCl_3_ as the solvent. Thermal properties were analyzed using DSC and TGA (see Supporting Information).

## 3 Results and Discussion

The synthesis involves the selective hydrogenation of cardanol to a cyclohexanone derivative bearing a long alkyl substituent, followed by a Baeyer–Villiger oxidation (BVO) to yield the corresponding lactone. The BVO of the cyclohexanone derivative is intended to be carried out under Mukaiyama conditions, which, to date, has not yet been reported. Compared to the use of reagents such as *m*CPBA, this approach offers a significantly lower safety risk, particularly when scaling up to larger quantities, in addition to the use of abundant oxygen as green oxidizer. Subsequently, the polymerization of this lactone has been investigated, since the incorporation of this lactone into the backbone of conventional PCL is of great interest for gaining novel, unique polymer properties.

The first step in the synthetic pathway is the selective hydrogenation of cardanol to the corresponding ketone. Palladium catalysts offer high selectivities toward ketone during phenol hydrogenation; thus, Pd/C is employed as a commercially available catalyst [18]. To optimize reaction conditions, the hydrogen pressure was systematically varied during the hydrogenation step (Figure 2).

**FIGURE 2:**
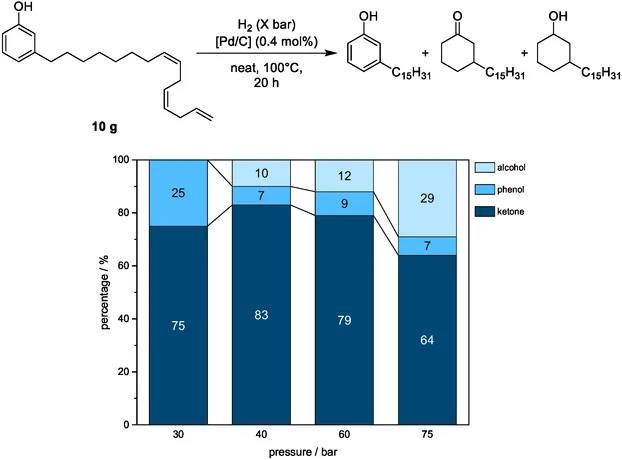
Product mixture composition in relation to hydrogen pressure.

The reaction was carried out under neat conditions on a 10 g scale. It was demonstrated that the optimal hydrogen pressure is 40 bar, under which 83% ketone was obtained. Applying higher pressures did not enable complete hydrogenation without residual phenol. Instead, it only increased the proportion of alcohol in the product mixture. These obtained product formation results exceed previously reported hydrogenations of cardanol, where yields of ketone derived from cardanol typically range between 70% and 72% [16, 17, 19]. Moreover, unlike the literature procedures that rely on solvent systems such as hexane or cyclohexane, the present method operates efficiently under neat conditions.

However, from a conceptual perspective, an attractive pathway would be the hydrogenation at 30 bar. Here, only a conversion of 75% is achieved, but the remaining phenol derivative could be recycled back into the production process, whereas the resulting cyclohexanol derivative after over‐hydrogenation cannot be directly recycled. Furthermore, it was of interest to look at the kinetics of the hydrogenation under neat conditions (Figure 3). This reaction was carried out at a hydrogen pressure of 75 bar.

**FIGURE 3:**
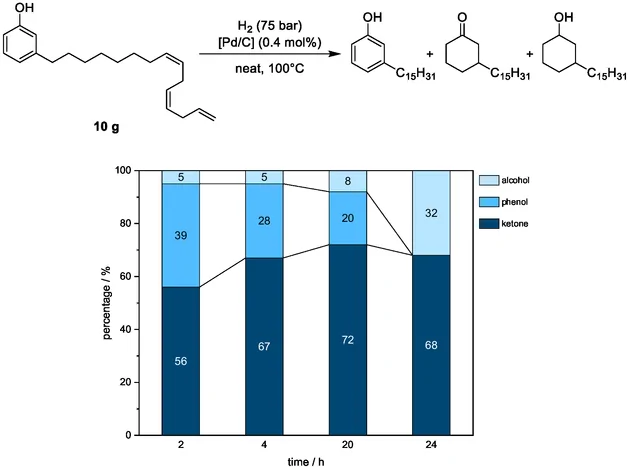
Kinetics of the cardanol hydrogenation.

It was shown that complete hydrogenation of cardanol can be achieved after 24 h, yielding 68% of the ketone. However, at 20 h, the selectivity toward the ketone was higher (72% of ketone), with 20% phenol remaining. This suggests that in the later stages of the reaction, the conversion of the ketone to the alcohol occurs at a rate equal to or faster than the conversion of phenol to the ketone. Therefore, stopping the reaction after 20 h is preferable, since the remaining phenol could be recovered and recycled.

The separation of the ketone out of the reaction mixture was of great importance and was initially achieved using both normal‐phase (NP) and reverse‐phase (RP) column chromatography (NP: cyclohexane:ethyl acetate 9:1; RP: acetonitrile, 0.1% acetic acid). The use of both chromatographic methods was necessary due to insufficient separation achieved with the applied automated column. It was further observed that increasing the column loading led to a band broadening of the substances between fractions. Because larger amounts of lactone in the multi‐10 g range were desired to be synthesized in order to generate sufficient lab sample quantities for the subsequent polymerization studies, further chromatographic purification was avoided due to the large volumes of solvent required. Therefore, we looked for alternative separation methods that are simpler, faster, and less resource intensive.

A basic workup followed by extraction of the reaction mixture would be a conceivable approach, whereby the phenol derivative would be transferred to the aqueous phase, while the ketone and alcohol would remain in the organic phase. Accordingly, the crude product was treated with KOH in ethanol (2 M), which resulted in a yellow coloration of the mixture. This can be attributed to the deprotonation of the phenol derivative. The reaction mixture was then extracted with DCM (3 × 20 mL), and the combined organic phases were washed with water and dried over sodium sulfate. However, after solvent removal, the presence of the phenol derivative was still detected. Simple deprotonation of the phenol may not be sufficient, likely due to its surfactant‐like properties. Alternatively, the phenol derivative could be chemically modified using succinic anhydride to yield a carboxylic acid. It was demonstrated that complete conversion of the phenol can be achieved after 17 h (Scheme 1).

**SCHEME 1:**
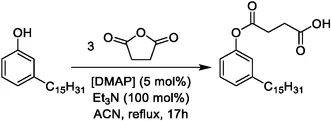
Conversion of phenol derivate to the respective carboxylic acid with succinic anhydride.

Additionally, it was proven that the ketone component was not affected by the reaction conditions, which is paramount if a good yield for the ketone is to be obtained (see Table S6). The obtained carboxylic acid salt could then be filtered off using basic alumina, while the ketone was obtained in 99% purity. No presence of the substituted cyclohexanol or phenol derived from cardanol was detected using routine ^1^H NMR spectroscopy.

Next to this, an alternative isolation method was found, which consists of a simple treatment of the crude reaction mixture with triethylamine, leading to the formation of the ammonium salt of the substituted phenol. This salt could then be separated by filtration over basic alumina, allowing the ketone to be obtained in 85% yield. One reason for the efficient separation achieved by filtration is the ability of ammonium ions to form hydrogen bonds with the stationary phase. The ammonium phenol salt interacts strongly with the basic alumina, which significantly retards its passage through the filter column. In contrast, as previously described, the formation of a sodium or potassium salt of the phenol then leads to a lack of capacity for hydrogen bonding. Like the substituted phenol, the corresponding cyclohexanol derived from cardanol is also retained on the column material, which is rationalized by acid–base interactions of the alcohol moiety with the basic alumina stationary phase. These interactions are weaker than those of the phenol but stronger than those of the ketone [20]. They are nonetheless strong enough to separate both the phenolic and the alcoholic species from the ketone.

To the best of our knowledge, this developed methodology has not been previously reported in the literature. To date, three procedures for the hydrogenation of cardanol have been described, all of which appear difficult to be applied on an industrial scale [21]. The first, by Lemaire et al. [19], involves purification by column chromatography followed by recrystallization from ethanol, which affords only moderate yields of 70% and a purity of just 90% (Scheme 2).

Additionally, conversion of the alcohol with phenyl isocyanate has been reported by Deutsch et al. [17], in which the resulting carbamate is separated during distillation. However, this method requires the addition of stoichiometric amounts of isocyanate and is therefore suitable only for laboratory‐scale applications (Scheme 2).

**SCHEME 2:**
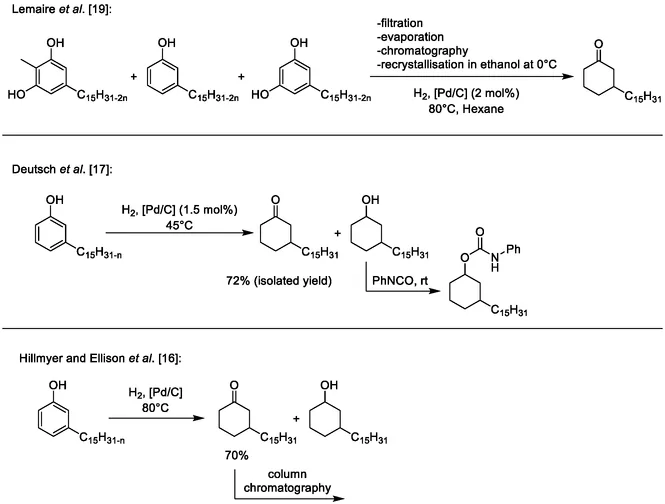
Reaction procedures according to Lemaire et al. [19], Deutsch et al. [17], and Hillmyer and Ellison et al. [16].

In a study by Hillmyer and Ellison et al. [16], the ketone was also prepared via hydrogenation of cardanol, with the ketone being solely purified using column chromatography.

In comparison, the method applied in this work achieves an 85% isolated yield with 99% purity with significantly less experimental effort—and yields a product that can directly be employed for polymerization (see below). Although we consider such a column filtration to be suitable for technical purposes too, at large scale a distillation might represent a more favored and efficient approach to purify the ketone product derived from cardanol. In addition, optimization of the hydrogenation process in order to gain increased selectivity for this ketone is a further future research task, and such an increased selectivity would then also simplify the downstream processing and product isolation.

The second reaction step involves the BVO of the obtained ketone to yield the corresponding lactones. This transformation can be carried out either via the classical BVO, employing hydrogen peroxide or a preformed peracid, or alternatively by introducing an aldehyde as a cosubstrate in combination with molecular oxygen as an oxidation reagent, which then generates the peracid in situ [22]. For the synthesis of the reference lactone compound, the ketone was subjected to reaction with *m*CPBA, as illustrated in Scheme 3, similar to the method described by Hillmyer and Ellison et al. [16].

**SCHEME 3:**
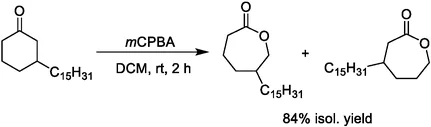
Synthesis of PDCL from the corresponding ketone.

**FIGURE 4:**
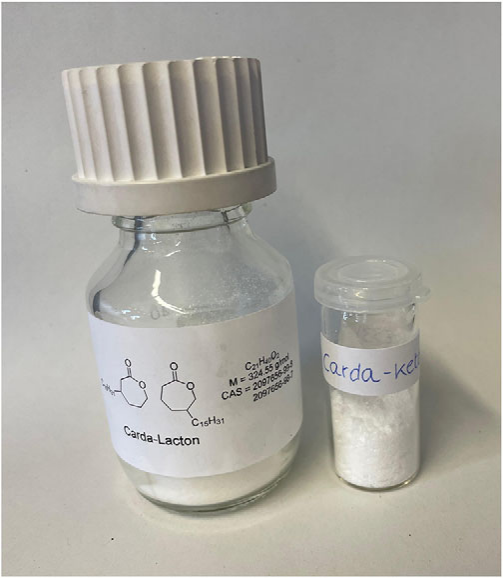
Lactone product (“carda‐lactone”) on the left and the corresponding ketone intermediate (“carda‐ketone”) on the right.

Full conversion to the lactones (PDCL) was achieved after 2 h of reaction time at room temperature. The workup was carried out by basic washing with sodium hydroxide solution, affording a yield of 84% (Figure 4). The regioselectivity of the BVO was determined via a ring‐opening reaction with benzyl alcohol, as the characteristic NMR signals of the two lactone regioisomers overlapped (Scheme 4).

**SCHEME 4:**
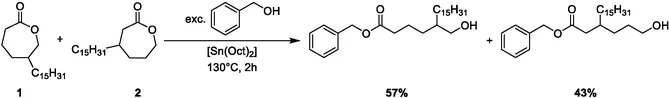
Ring‐opening reaction of PDCL with benzyl alcohol.

Full conversion and suppression of polymerization were achieved by employing an excess of benzyl alcohol. Under these conditions, a regioisomeric ratio of 57:43 in favor of lactone **1** was observed. This outcome is expected, as the alkyl side chain is not in close proximity to the migrating group and therefore should not significantly influence the regioselectivity of the reaction.

Alternatively, the BVO can be performed by adding an aldehyde and employing molecular oxygen as the oxidant. This approach, known as the Mukaiyama method, has been applied to a variety of ketones, most notably cyclohexanone [23, 24, 25, 26]. However, this synthetic strategy has not yet been explored for this particular substituted cyclohexanone, and its implementation could offer additional advantages when developing a technical process based on this methodology.

The primary reason for the rigorous purification described previously lies in the radical mechanism of the BVO when using an aldehyde as the cosubstrate. Any residual phenol present in the reaction mixture can inhibit both the formation and transfer of radicals, thereby significantly retarding the reaction. As a result, the oxidation of the aldehyde, and consequently the oxidation of the cyclohexanone, cannot proceed efficiently.

In an initial experiment, the effect of the aldehyde amount on the conversion was examined. Benzaldehyde was selected as the model cosubstrate, as it is the preferred aldehyde reported in the literature for the Mukaiyama system (Figure 5) [27].

**FIGURE 5:**
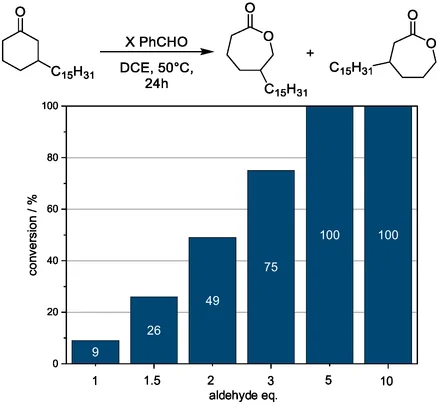
Conversion to lactones in relation to amount of benzaldehyde used.

It was observed that full conversion is achieved when 5 equivalents of benzaldehyde are used. To reduce the required amount of benzaldehyde, the aldehyde can be added gradually over time, thereby maintaining a low aldehyde concentration throughout the reaction. This approach increases the relative concentration of the ketone compared to the aldehyde, resulting in improved conversion to the lactone. This strategy has been demonstrated successfully by us in a previous publication concerning the oxidation of cyclohexanone to *ε*‐caprolactone using aliphatic aldehydes as cosubstrates [28]. Subsequently, the influence of the solvent was investigated (Figure 6).

**FIGURE 6:**
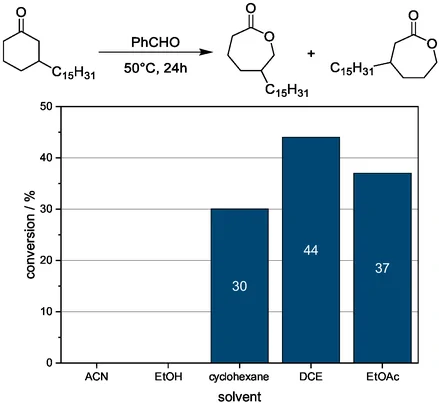
Influence of solvent on the BVO of carda‐ketone with benzaldehyde as cosubstrate.

It was observed that dichloroethane is the most effective solvent, achieving approximately 44% conversion. Ethyl acetate also provided good conversions. In contrast, ethanol and acetonitrile did not result in any conversion. This is consistent with literature reports, which indicate that protic solvents such as ethanol are ineffective in the BVO under Mukaiyama conditions, owing to their ability to act as competing nucleophiles and their propensity to quench radical intermediates [29].

Finally, the nature of the aldehyde cosubstrate was investigated. Ideally, the carboxylic acid byproduct should add economic value relative to the starting aldehyde. Aliphatic aldehydes were therefore also tested, as they are large‐volume industrial products synthesized via the efficient hydroformylation of olefins [30].

When comparing different aldehydes (Figure 7), it is evident that benzaldehyde affords the highest conversion to the two lactones, achieving 86%. In contrast, aliphatic aldehydes such as butyraldehyde and valeraldehyde result in significantly lower conversions, both below 50%. This can be attributed to the electronic structure of the peracid formed in situ. Notably, however, the difference in conversion between benzaldehyde and the aliphatic aldehydes is less pronounced in this case compared to the analogous reaction with cyclohexanone [27, 28], suggesting that the ketone derived from cardanol reacts more favorably with aliphatic peracids compared to cyclohexanone.

**FIGURE 7:**
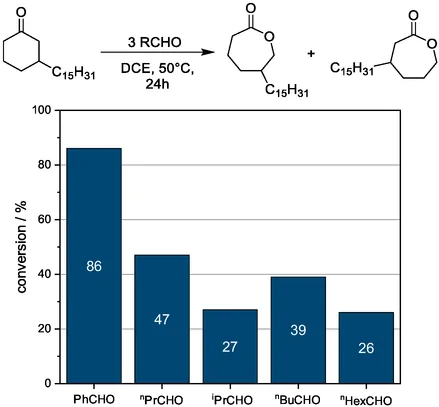
Different aldehydes used for the BVO of carda‐ketone.

### 3.1 Homo‐ and Copolymerization of PDCL

The thus prepared PDCL was subsequently subjected to oligo‐ and polymerizations (Figure 8). All reactions in the following were conducted using organocatalysts, namely, TBD and 1,8‐diazabicyclo [5.4.0]undec‐7‐ene (DBU), which are bicyclic guanidine‐ and amidine‐type organobases, respectively (Figure 9). They can also be classified as some of the gentler superbases (p*K*_bH_^+^ = 24.3 (DBU) and 26.0 (TBD), measured in acetonitrile [31]) and differ in that TBD is known for its clear‐cut bifunctional behavior, while DBU typically acts as a Brønsted base [32, 33]. Thus, to efficiently polymerize lactone monomers, DBU typically needs the support of a cocatalyst, as, for example, 1‐[3,5‐bis(trifluoromethyl)phenyl]‐3‐cyclohexylthiourea (TU) [34, 35].

**FIGURE 8:**
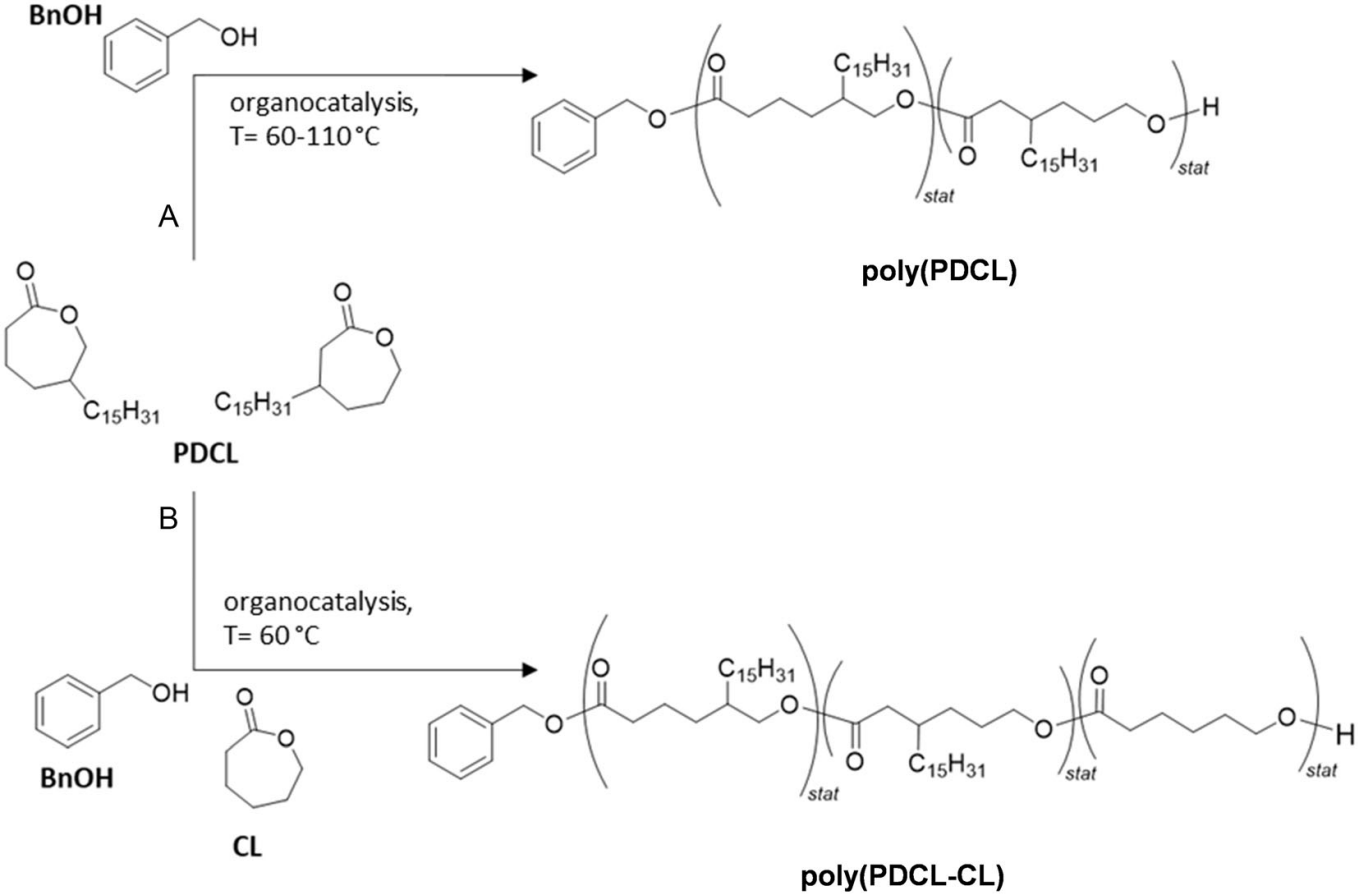
Scheme of the homo‐ (A) and co‐polymerization (B) of PDCL conducted in this work.

We also opted for bulk polymerizations instead of a solvent‐based process (e.g., DCM), on account of the benefits of solvent‐free approaches regarding sustainability and scale‐up.

For bulk polymerizations, higher temperatures are necessary, as PDCL is solid at room temperature. Thus, in a first set of reactions, at 60 and 110 °C, respectively, polymerization was successful both with TBD or DBU (Figure 10). Using the former, high conversions are reached at 60 and 110 °C. In comparison, DBU‐catalyzed reactions deliver lower conversions of 25% at 60 °C and 71% at 110 °C, respectively. Note that in all cases above, oligomeric structures were targeted, and relatively high loadings of catalyst were employed (10 mol% relative to monomer). Nonetheless, these preliminary results underlined that the PDCL received from the synthetic process outlined in the previous section is ready to be directly converted into a short‐chain polymer. However, the subsequent polymerizations employed PDCL, which was additionally filtered over basic alumina before use, resulting in enhanced conversions, most likely because traces of phenol were removed. At 110 °C, molar mass distributions (via GPC) of *Ð*_M_ = 2.2 and 1.7 were found for the application of TBD and DBU, respectively.

**FIGURE 9:**
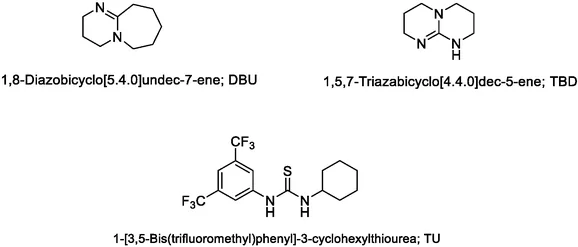
Catalysts and cocatalyst employed for the homo‐ and copolymerization of PDCL.

A closer look at the polymerization kinetics (Figure 11) of the reactions using TBD revealed that at 60 °C, near‐quantitative conversion (97%) is reached after 60 min, following a well‐defined first‐order kinetics in monomer consumption (Figure S3). At 110 °C, already after 15 min, 98% conversion is found, clearly highlighting that TBD is an effective catalyst under these conditions. The fast monomer consumption also explains why a relatively broad molar mass distribution is observed after 24 h of reaction time, suggesting considerable transesterification occurs once the monomer has been largely consumed.

**FIGURE 10:**
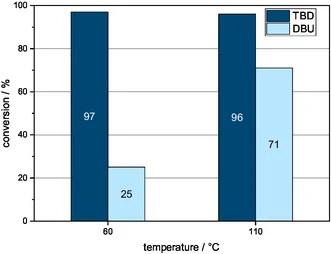
Polymer conversions at different temperatures using TBD and DBU as the catalyst. Conditions: PDCL/catalyst/BnOH = 50:2:5 (molar), bulk, 24 h.

Based on these findings, optimized conditions were applied, and higher molar masses were targeted. Using 200 eq. of the PDCL monomer mixture, 2 eq. benzyl alcohol, and 5 eq. TBD (2.5 mol% catalyst loading), high conversions of 94% were obtained after 4 h at 60 °C (Figure 12). The resulting polyester displayed a molar mass of *M*_n_ = 6.6 × 10^3^ g/mol (*Ð*_M_ = 2.01) as determined via GPC measurement (CHCl_3_, polystyrene calibration). This result is notably lower than the calculated average molecular weight of 29.6 × 10^3^ g/mol derived from the ratio of repeating units versus initiator via ^1^H NMR. This deviation can be attributed to three factors. First, this type of ROP is sensitive to transesterification [36], which can occur by intra‐ and intermolecular reactions, forming cyclic or linear polyesters, respectively. Transesterification will entail a broadening of polydispersity and a lowering of *M*_n_. The observed molar mass distribution in this work (*Ð*_M_ = 1.7–2.2) and the typical reaction conditions (bulk) both suggest transesterification as a major contributor to the observed low poly(PDCL) molar masses. Second, the presence of trace amounts of water can be assumed. The monomer is used as is or after minimal purification (filtering over basic alumina) to mimic technical processes where extensive monomer drying is unrealistic. Water is not fully removed this way, and its presence will lower the actual initiator to monomer ratio, hence entailing lower polyester molar masses. Finally, it must be considered that GPC will deliver only relative polymer masses and might underestimate the *M*_n_ of this type of less‐polar aliphatic polyester [37].

**FIGURE 11:**
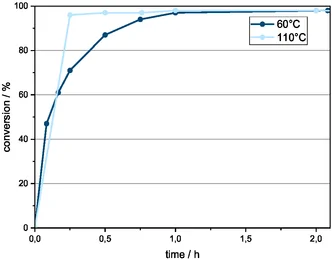
Kinetic measurements for TBD‐catalyzed polymerization at 60 and 110 °C. Conditions: PDCL/TBD/BnOH = 50:2:5 (molar), bulk.

Thermal properties of the received poly(PDCL) were determined using TGA and DSC. TGA analysis revealed a 10% mass loss at 367 °C, while full thermal decomposition was recorded at 479 °C. Hence, the thermal stability of poly(PDCL) is somewhat superior to that of poly(*ε*‐caprolactone) (PCL) [38, 39]. DSC analysis (Figure 13) revealed a clear melting endotherm at 26.9 °C in both the first and second heating cycles. The melting enthalpy was determined to be Δ*H*_m_ = 15.8 J·g^−1^ (second heating cycle), while the recrystallization exotherm was found at 17.1 °C with Δ*H*_c_ = −10.9 J·g^−1^.

**FIGURE 12:**
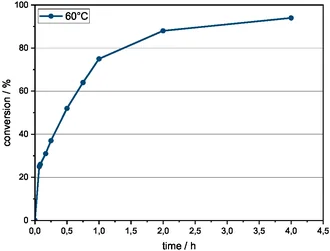
TBD‐catalyzed polymerization of PDCL. Conditions: PDCL/TBD/BnOH = 200:5:2, bulk, 60 °C.

The observed melting endotherm was attributed to side‐chain crystallization (C_15_‐alkyl chains as pendant groups), which is in accordance with findings by Hillmyer and coworkers [16]. It should be noted that main‐chain crystallization of poly(PDCL) is hindered by several factors. For one, as discussed in the previous section, PDCL itself is a mixture of structural isomers (carrying the alkyl chain in *β*‐ or *δ*‐position, respectively) so that poly(PDCL) is most accurately described as a statistical copolyester of these two isomers. Furthermore, the ring‐opening polymerization as described in this work is not stereoselective, so in addition to irregular spacing of the pending alkyl chains, also an atactic polymer results. In principle, such a feature recommends PDCL as a potential comonomer to endow otherwise semicrystalline oligoesters or polyesters (such as the widely employed poly(*ε*‐caprolactone) [40, 41]) with modulated or even fully suppressed crystallinity, as its incorporation should effectively disrupt the ability of the copolyester chains to organize in crystalline structures. Such a feature would be highly desirable, since currently often expensive and fossil‐based comonomers, such as δ‐valerolactone (VL), are employed to this effect [42]. As a proof of principle, CL, the homopolymer of which is semicrystalline with a well‐defined melting point at *T*_m_ = 54 °C (Table 1, entry 1) [43], and PDCL were copolymerized in one‐pot batch polymerizations. Optimized reaction conditions were inferred from the above results for PDCL homopolymerization.

**TABLE 1:** Summary of DSC thermal transitions for copolymers PDCL/CL with varying monomer ratios, including melting (*T*_m_), crystallization (*T*_c_), and corresponding enthalpy changes (Δ*H*_m_, Δ*H*_c_). Reaction conditions: PDCL + CL/TBD/TU/BnOH = 100:1.25:1.25:1.25, bulk, 1 h, 60 °C.

Entry	PDCL:CL^a^	*T*_10% decomposition_, °C	*T*_m_, °C	Δ*H*_m_, J g^−1^	*T*_c_, °C	Δ*H*_c_, J g^−1^
1	0:100	292	54	12	26	12
2	10:90	351	49	8.5	−2	9.0
3	30:70	303	16	1.9	7	1.8
4	50:50	354	14	10.2	7	10.5
5	70:30	296	19	11.3	11	10.4
6	100:0	367	27	15.8	17	10.9

a Total molecular equivalents of monomers (100 eq.) in comparison to the Lewis base (1.25 eq.).

Thus, initial syntheses were carried out at 60 °C, utilizing a 50:50 monomer ratio (molar) whereby 2.5 mol% catalyst loading (TBD) was employed. Complete conversion of both monomers was achieved after 45 min. For a more controlled polymerization, the addition of 2.5 mol% TU as a cocatalyst was investigated. In this case, fast monomer consumption (97% conversion after 45 min) was accompanied by a moderate narrowing of the molar mass distribution (*Ð*_M_ = 1.8 for TBD vs. 1.6 for TBD/TU), while molar masses remained comparable (1.7 × 10^4^ and 1.6 × 10^4^ g/mol, respectively, see Figure S5 for GPC traces).

**FIGURE 13:**
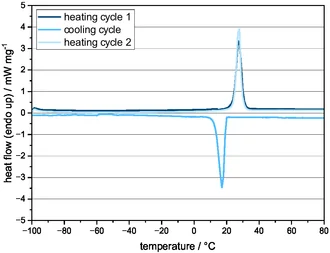
DSC thermogram of poly(PDCL). Heating rate = 10 K·min^−1^, range from −100 to 200 °C.

After these initial investigations, the catalyst loading was decreased to 1.25 mol% and three catalyst systems (TBD, TBD/TU, and DBU/TU) were compared. TBD/TU displayed the best performance, yielding 100% conversion after 75 min (Figure 14). TBD delivered 75% under identical conditions, while for DBU/TU a 29% conversion was found (Figure 14).

**FIGURE 14:**
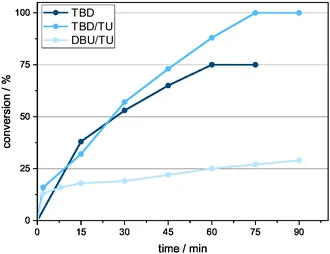
Copolymerization kinetics using different catalytic systems (overall monomer conversion). Conditions: PDCL/CL/TBD/TU/BnOH = 50:50:1.25:1.25:1.25 (molar), bulk, 60 °C.

The microstructure of the resulting copolymer samples was analyzed via NMR measurements. DOSY ^1^H NMR analysis showed that all resonances observed in the ^1^H NMR spectrum exhibited the same diffusion coefficient (*D*  = 1.4 × 10^−6^ cm^2^ s^−1^, see Figures S19 and S21), suggesting that the signals arise from a single macromolecular species and indicating successful copolymer formation with no residual homopolymer present. Dyad analysis [44] of the carbonyl region of the resulting comonomer sequences (for ^13^C NMR measurement, see Figure S20) also indicates a statistical copolyester.

This is further supported by a qualitative analysis of these dyads after specific polymerization time intervals. A consistent incorporation rate of PDCL and CL (see details in Figure S22) is observed. This substantiates a statistical arrangement in the copolyester, since no monomer is enchained at a much faster rate than the other, as would have been the case for a block‐like copolymeric structure.

The thermal properties (Table 1 and Figure S6) of copolymers prepared from different ratios of the comonomers are also in full accordance with a statistical polymer microstructure. For example, the 50:50 (PDCL:CL) copolymer, as determined via DSC, exhibited a single melting point below those of the individual homopolymers (Table 1, entry 4), while the 10:90 (PDCL:CL) copolymer showed a melting point close to that of PCL (Table 1, entries 1 and 2). The 70:30 (PDCL:CL) copolymer displayed a melting signal comparable to the PDCL homopolymer (Table 1, entries 5 and 6). Interestingly, the polyester resulting from a ratio of 30:70 is almost fully amorphous, displaying only very minor traces of remaining crystallinity (Table 1, entry 3, and Figure S6b) with a very low Δ*H*_m_ = 1.9 J g^−1^ and a melting point below room temperature. This clearly recommends PDCL as a biobased comonomer for tailoring copolyester properties, such as room‐temperature liquid additives for coatings and paints.

TGA of the 50:50 (PDCL:CL) copolymer shows full decomposition at 466 °C and 10% mass loss at 354 °C.

## 4 Conclusion

The development of polymers that are both biodegradable and biobased represents a major challenge and opportunity for the chemical industry in the coming years. In this study, we presented a straightforward and potentially scalable synthesis of a cardanol‐derived lactone (which is obtained as a mixture of two regioisomers) and investigated its homopolymerization as well as copolymerization with *ε*‐caprolactone. Although PDCL has been previously described, neither a larger sample preparation together with a potentially industrially viable synthesis route nor its (co)polymerization using organocatalysts has been reported until now.

Starting from cardanol, we achieved selective partial hydrogenation to the corresponding ketone with 83% selectivity at 40 bar hydrogen pressure. Complete suppression of further hydrogenation was not observed. We introduced a novel practical workup procedure, where after adding triethylamine, the crude product was filtered over basic alumina to efficiently remove residual phenol and alcohol derivatives. This approach yielded the product in 85% yield and with 99% purity, surpassing previously reported methods.

The BVO of the ketone was then performed using two approaches: the conventional *m*CPBA method and the Mukaiyama method with an aldehyde cosubstrate. Notably, this type of oxidation has not been previously reported for this ketone. Using benzaldehyde as the cosubstrate, complete conversion to the lactone was achieved after 24 h at 50 °C with 5 eq. of aldehyde. This Mukaiyama method is particularly promising for large‐scale purpose in cases when the resulting carboxylic acid byproduct can add value, thus enhancing the economic viability of the process. A suitable aldehyde would be one of lower economic value than its corresponding carboxylic acid. Representative examples of such aldehydes are *n*‐butyraldehyde and isobutyraldehyde. In continuation of this research work as well as our recently reported BVO process based on this Mukaiyama method for the preparation of ε‐caprolactone [28], developing further and scaling up this coupled approach for the synthesis of the cardanol‐derived lactone regioisomer mixture in order to demonstrate its industrial viability is the goal of future work.

Finally, we demonstrated the successful homopolymerization and copolymerization of the thus‐isolated lactone regioisomer mixture. Metal‐free homopolymerization can be readily achieved using a commercial guanidine‐type catalyst (TBD). The poly(PDCL) received this way displays moderate crystallinity, most likely originating from side‐chain crystallization, as was observed previously [16]. Importantly, the copolymerization with CL was successful, resulting in a statistical incorporation of the PDCL monomer. Accordingly, crystallinity and melting point of the obtained copolyester can be tailored by controlling the ratio of PDCL to CL. About 30% of PDCL incorporation entails a largely amorphous copolymer with a melting point below room temperature, suggesting a high potential as a bio‐based or partly bio‐based alternative to fossil‐derived polyester additives to coatings, paints, and similar applications. Future improvements of the polymerization procedure should address the realization of higher molar masses and a reduction of transesterification, especially in view of the desired solvent‐free and elevated temperature conditions.

## Funding

This study was supported by Federal Ministry of Education and Research (Bundesministerium für Bildung und Forschung, BMBF), now Federal Ministry of Research, Technology and Space (Bundesministerium für Forschung, Technologie und Raumfahrt, BMFTR) (Grant 031B1140CX).

## Conflicts of Interest

P.R.W. and H.G. are inventors of a filed patent application that covers findings described in this article. All the other authors declare no conflicts of interest.

## Supporting information

Supplementary Material

## Data Availability

The data that support the findings of this study are available in the supporting information of this article.

## References

[1] S. Ranganathan , S. Dutta , J. A. Moses , and C. Anandharamakrishnan , “Utilization of Food Waste Streams for the Production of Biopolymers,” Heliyon 6, no. 9 (2020): e04891. 10.1016/j.heliyon.2020.e04891 PMC7502569 32995604

[2] T. Seidensticker , A. J. Vorholt , and A. Behr , “The Mission of Addition and Fission—Catalytic Functionalization of Oleochemicals,” European Journal of Lipid Science and Technology 118, no. 1 (2016): 3.

[3] M. von Czapiewski and M. A. R. Meier , “Catalytic Oxyfunctionalization of Methyl 10‐Undecenoate for the Synthesis of Step‐Growth Polymers,” Macromolecular Chemistry and Physics 218, no. 16 (2017): 1700153.

[4] S. Caillol , “The Future of Cardanol as Small Giant for Biobased Aromatic Polymers and Additives,” European Polymer Journal 193 (2023): 112096.

[5] A. Parambath , Cashew Nut Shell Liquid. A Goldfield for Functional Materials (Springer, 2017).

[6] J. H. P. Tyman , “Non‐Isoprenoid Long Chain Phenols,” Chemical Society Reviews 8, no. 4 (1979): 499, 10.1039/CS9790800499.

[7] J. Valette , “Improvement of Extraction Methods of Malian Cashew Nut Shell Liquid,” SDRP Journal of Earth Sciences & Environmental Studies 6 (2022): 121.

[8] Polaris Market Research , Cashew Nutshell Liquid Market Share, Size, Trends, Industry Analysis Report, By Product (Surfactants, Epoxy Curing Agents, Polyols & NCO Blocking Agents, Epoxy Modifiers & Resins, Phenol Formaldehyde Resins, Others). By Application; By Region; Segment Forecast, 2022–2030, 2022.

[9] G. John , M. Masuda , Y. Okada , K. Yase , and T. Shimizu , “Nanotube Formation From Renewable Resources via Coiled Nanofibers,” Advanced Materials 13, no. 10 (2001): 715, 10.1002/1521-4095(200105)13:103.0.CO;2-Z.

[10] V. S. Balachandran , S. R. Jadhav , P. K. Vemula , and G. John , “Recent Advances in Cardanol Chemistry in a Nutshell: From a Nut to Nanomaterials,” Chemical Society Reviews 42, no. 2 (2013): 427, 10.1039/c2cs35344j. 23114456

[11] A. H. Tullo , “A Nutty Chemical,” Chemical & Engineering News Archive 86, no. 45 (2008): 26.

[12] A. Anbazhagan , P. R. Choudhury , J. L. Saraswathi , and S. Sambandam , “Sustainable Composite of Cardanol Based Phenalkamine Cured Epoxy Systems: Fabrication, Characterization and Mechanical Performance Evaluation for Emerging Applications,” Journal of Polymer Materials 41, no. 4 (2024): 299.

[13] C. K. S. Pillai , V. S. Prasad , J. D. Sudha , S. C. Bera , and A. R. R. Menon , “Polymeric Resins From Renewable Resources. II. Synthesis and Characterization of Flame‐Retardant Prepolymers From Cardanol,” Journal of Applied Polymer Science 41, no. 9–10 (1990): 2487, 10.1002/app.1990.070410947.

[14] A. Kumar , P. K. Vemula , P. M. Ajayan , and G. John , “Silver‐Nanoparticle‐Embedded Antimicrobial Paints Based on Vegetable Oil,” Nature Materials 7, no. 3 (2008): 236, 10.1038/nmat2099. 18204453

[15] S. Shukla , A. Mahata , B. Pathak , and B. Lochab , “Cardanol Benzoxazines—Interplay of Oxazine Functionality (Mono to Tetra) and Properties,” RSC Advances 5, no. 95 (2015): 78071.

[16] H. J. Kim , K. Jin , J. Shim , W. Dean , M. A. Hillmyer , and C. J. Ellison , “Sustainable Triblock Copolymers as Tunable and Degradable Pressure Sensitive Adhesives,” ACS Sustainable Chemistry & Engineering 8, no. 32 (2020): 12036.

[17] J. Deutsch and A. Köckritz , “Synthesis of Novel Chemicals From Cardanol as a Product of Cashew Nutshell Processing,” Food Science & Nutrition 8, no. 7 (2020): 3081. 10.1002/fsn3.1480 PMC7382122 32724572

[18] S. Nishimura , Handbook of Heterogeneous Catalytic Hydrogenation for Organic Synthesis (John Wiley & Sons, Inc, 2001).

[19] A. I. Rahobinirina , M. F. Rakotondramanga , A. Berlioz‐Barbier , E. Métay , V. Ramanandraibe , and M. Lemaire , “Valorization of Madagascar's CNSL via the Synthesis of One Advanced Intermediate (3‐Pentadecylcyclohexanone),” Tetrahedron Letters 58, no. 23 (2017): 2284.

[20] S. Cai and K. Sohlberg , “Adsorption of alcohols on γ‐alumina (1 1 0 C),” Journal of Molecular Catalysis A: Chemical 193, no. 1–2 (2003): 157, 10.1016/S1381-1169(02)00457-0.

[21] R. A. Sheldon , “Metrics of Green Chemistry and Sustainability: Past, Present, and Future,” ACS Sustainable Chemistry & Engineering 6, no. 1 (2018), 10.1021/acssuschemeng.7b03505.

[22] M. Renz and B. Meunier , “100 Years of Baeyer–Villiger Oxidations,” European Journal of Organic Chemistry 1999, no. 4 (1999): 737.

[23] Y.‐F. Li , M.‐Q. Guo , S.‐F. Yin , et al., “Co_3_O_4_ of Regular Cubic Shape as High‐Efficiency Catalyst for the Preparation of Lactones Through the Baeyer–Villiger Oxidation of Cyclic Ketones With Dioxygen,” Reaction Kinetics, Mechanisms and Catalysis 109, no. 2 (2013): 525.

[24] X. Li , F. Wang , H. Zhang , C. Wang , and G. Song , “Baeyer‐Villiger Oxidation of Cyclohexanone With Molecular Oxygen in the Presence of Benzaldehyde,” Synthetic Communications 26, no. 8 (1996): 1613.

[25] S.‐I. Murahashi , Y. Oda , and T. Naota , “Fe_2_O_3_‐Catalyzed Baeyer‐Villiger Oxidation of Ketones With Molecular Oxygen in the Presence of Aldehydes,” Tetrahedron Letters 33, no. 49 (1992): 7557.

[26] T. Yamada , K. Takahashi , K. Kato , T. Takai , S. Inoki , and T. Mukaiyama , “The Baeyer–Villiger Oxidation of Ketones Catalyzed by Nickel(II) Complexes With Combined Use of Molecular Oxygen and an Aldehyde,” Chemistry Letters 20, no. 4 (1991): 641.

[27] H.‐Y. Lan , X.‐T. Zhou , and H.‐B. Ji , “Remarkable Differences Between Benzaldehyde and Isobutyraldehyde as Coreductant in the Performance Toward the Iron(III) Porphyrins‐Catalyzed Aerobic Baeyer–Villiger Oxidation of Cyclohexanone, Kinetic and Mechanistic Features,” Tetrahedron 69, no. 21 (2013): 4241.

[28] P. R. Wohlfahrt and H. Gröger , “Efficient Combination of Two Industrial Processes for Sustainable Production of ε‐Caprolactone,” Industrial & Engineering Chemistry Research 65, no. 5 (2026): 2454.

[29] N. Mora‐Diez , S. Keller , and J. R. Alvarez‐Idaboy , “The Baeyer‐Villiger Reaction: Solvent Effects on Reaction Mechanisms,” Organic & Biomolecular Chemistry 7, no. 18 (2009): 3682. 10.1039/b906058h 19707672

[30] J. Kubitschke , H. Lange , and H. Strutz , “Carboxylic Acids, Aliphatic,” in Ullmann's Encyclopedia of Industrial Chemistry (Wiley‐VCH Verlag GmbH & Co. KGaA, 2000), ISBN 9783527306732.

[31] I. Kaljurand , A. Kütt , L. Sooväli , et al., “Extension of the Self‐Consistent Spectrophotometric Basicity Scale in Acetonitrile to a Full Span of 28 p*K* _a_ Units: Unification of Different Basicity Scales,” The Journal of Organic Chemistry 70, no. 3 (2005): 1019. 10.1021/jo048252w 15675863

[32] J. G. Kim , G. S. Lee , and A. Lee , “Triazabicyclodecene: A Versatile Catalyst for Polymer Synthesis,” Journal of Polymer Science 62, no. 1 (2024): 42.

[33] R. C. Pratt , B. G. G. Lohmeijer , D. A. Long , R. M. Waymouth , and J. L. Hedrick , “Triazabicyclodecene: A Simple Bifunctional Organocatalyst for Acyl Transfer and ring‐Opening Polymerization of Cyclic Esters,” Journal of the American Chemical Society 128, no. 14 (2006): 4556. 10.1021/ja060662+ 16594676

[34] B. G. G. Lohmeijer , R. C. Pratt , F. Leibfarth , et al., “Guanidine and Amidine Organocatalysts for Ring‐Opening Polymerization of Cyclic Esters,” Macromolecules 39, no. 25 (2006): 8574.

[35] O. I. Kazakov , P. P. Datta , M. Isajani , E. T. Kiesewetter , and M. K. Kiesewetter , “Cooperative Hydrogen‐Bond Pairing in Organocatalytic Ring‐Opening Polymerization,” Macromolecules 47, no. 21 (2014): 7463. 10.1021/ma501847x PMC4230384 25400295

[36] M. T. Martello , A. Burns , and M. Hillmyer , “Bulk Ring‐Opening Transesterification Polymerization of the Renewable δ‐Decalactone Using an Organocatalyst,” ACS Macro Letters 1, no. 1 (2012): 131, 10.1021/mz200006s. 35578491

[37] P. Olsén , T. Borke , K. Odelius , and A.‐C. Albertsson , “ε‐Decalactone: A Thermoresilient and Toughening Comonomer to Poly(L‐Lactide,” Biomacromolecules 14, no. 8 (2013): 2883, 10.1021/bm400733e. 23815125

[38] K. Thangunpai , D. Hu , X. Su , M. Kajiyama , M. A. Neves , and T. Enomae , “Thermal Stability of Polycaprolactone Grafted Densely With Maleic Anhydride Analysed Using the Coats‐Redfern Equation,” Polymers 14, no. 19 (2022): 4100. 10.3390/polym14194100 PMC9571202 36236052

[39] G. Sivalingam and G. Madras , “Thermal Degradation of Poly (ε‐Caprolactone,” Polymer Degradation and Stability 80, no. 1 (2003): 11.

[40] M. A. Woodruff and D. W. Hutmacher , “The Return of a Forgotten Polymer—Polycaprolactone in the 21st Century,” Progress in Polymer Science 35, no. 10 (2010): 1217.

[41] E. H. Backes , S. V. Harb , C. A. G. Beatrice , et al., “Polycaprolactone usage in Additive Manufacturing Strategies for Tissue Engineering Applications: A Review,” Journal of Biomedical Materials Research Part B: Applied Biomaterials 110, no. 6 (2022): 1479. 10.1002/jbm.b.34997 34918463

[42] R. F. Storey , K. R. Herring , and D. C. Hoffman , “Hydroxy‐Terminated Poly(ε‐Caprolactone‐Co‐δ‐Valerolactone) Oligomers: Synthesis, Characterization, and Polyurethane Network Formation,” Journal of Polymer Science Part A: Polymer Chemistry 29, no. 12 (1991): 1759.

[43] S. H. Murphy , G. A. Leeke , and M. J. Jenkins , “A Comparison of the use of FTIR Spectroscopy With DSC in the Characterisation of Melting and Crystallisation in Polycaprolactone,” Journal of Thermal Analysis and Calorimetry 107, no. 2 (2012): 669.

[44] S. Naumann and D. Wang , “Dual Catalysis Based on *N*‐Heterocyclic Olefins for the Copolymerization of Lactones: High Performance and Tunable Selectivity,” Macromolecules 49, no. 23 (2016): 8869.

