# Supplementary material for: Synthesis and Polymerization of a Lactone Building Block Derived From Cashew Nut Shells
Source: ChemSusChem. 2026 Oct 12;19(19):e71014. doi: 10.1002/cssc.71014 (PMC13656192; doi:10.1002/cssc.71014)
Supplement: Supplementary file 1 [file CSSC-19-e71014-s001.pdf]

Supporting Information

Synthesis and Polymerization of a Lactone Building Block Derived from Cashew Nut Shells

Paulo Wohlfahrt[1,‡], Martina Huber[2,‡], Kerstin Elsinghorst[1], Seraina Mihailescu[2], Hannah Cumming[2], Stefan Naumann[2,*] and Harald Gröger[1,*]

[‡] Shared first authorship

[1] Chair of Industrial Organic Chemistry and Biotechnology, Faculty of Chemistry, Bielefeld University, Universitätsstr. 25, 33615 Bielefeld, Germany

[2] Institute for Macromolecular Chemistry, Albert-Ludwigs-University of Freiburg, Stefan-Meier-Str. 31, 79104 Freiburg, Germany

*__Correspondence__: Prof. Dr. H. Gröger, Email: harald.groeger@uni-bielefeld.de; Prof. Dr. S. Naumann, E-mail: stefan.naumann@makro.uni-freiburg.de

Contents

1 Chemicals and standard working conditions ... 3

2 Analytics ... 5

2.1 Nuclear Magnetic Resonance ... 5

2.2 Gel permeation Chromatography ... 5

2.3 Differential scanning calorimetry & thermogravimetric analysis ... 6

2.4 Structural analysis of lactones ... 6

3 Synthesis ... 6

3.1 General experimental protocols ... 6

3.1.1 Selective Hydrogenation of Cardanol (GP1) ... 6

3.1.2 Ring-opening reaction of reaction mixture with succinic anhydride (GP2) ... 7

3.1.3 Baeyer-Villiger oxidation under Mukaiyama conditions (GP3) ... 7

3.1.4 Homo- and Copolymerisation of PDCL ... 7

3.2 Experimental protocols ... 8

3.2.1 Pressure screening of cardanol hydrogenation ... 8

3.2.2 Kinetics of cardanol hydrogenation ... 9

3.2.3 Purification of ketone ... 10

3.2.4 Baeyer-Villiger oxidation of carda-ketone with *m*CPBA ... 12

3.2.5 Baeyer-Villiger oxidation of carda-ketone under Mukaiyama conditions ... 13

3.2.6 Polymerisation of PDCL ... 15

4 NMR-Spectra 19

1 Chemicals and standard working conditions

All chemicals were purchased in the highest purity and used without further purification.

1,2-Dichloroethane (Fisher Scientific GmbH, Schwerte, Germany)

1,3,5-Trimethoxybenzene (BLD Pharmatech Ltd., Shanghai, China)

Acetonitrile (Honeywell Research Chemicals Seelze GmbH, Seelze, Germany)

Aluminum oxide 60 active basic (Merck Chemicals GmbH, Darmstadt, Germany)

Argon (Linde Gas, Bielefeld, Germany)

Benzaldehyde (KMF Laborchemie-Handels-GmbH, Cologne, Germany)

Benzyl alcohol (Sigma-Aldrich Chemie GmbH, Schnelldorf, Germany)

Butyraldehyde (Fisher Scientific GmbH, Schwerte, Germany)

Cardolite NX-2026 (Cardolite Corporation, Bristol, USA)

Cyclohexane (VWR International GmbH, Darmstadt, Germany)

Deuterated chloroform (Eurisotop, St.-Aubin Cedex, France)

4-Dimethylaminopyridine (BLD Pharmatech Ltd., Shanghai, China)

Ethanol (VWR International GmbH, Darmstadt, Germany)

Ethyl acetate (Fisher Scientific GmbH, Schwerte, Germany)

Heptanal (Alfa Aesar GmbH + Co. KG, Karlsruhe, Germany)

Heptane (Th. Geyer GmbH + Co. KG, Renningen, Germany)

Hydrogen (Linde Gas, Bielefeld, Germany)

Isobutyraldehyde (Fisher Scientific GmbH, Schwerte, Germany)

Isovaleraldehyde (Fisher Scientific GmbH, Schwerte, Germany)

meta-Chloroperbenzoic acid (Fisher Scientific GmbH, Schwerte, Germany)

Methanol (Fisher Scientific GmbH, Schwerte, Germany)

Nitrogen (Linde Gas, Bielefeld, Germany)

Oxygen (Linde Gas, Bielefeld, Germany)

Palladium on carbon (Sigma-Aldrich Chemie GmbH, Schnelldorf, Germany)

Poly-Caprolactone (Mn ~10,000 by GPC, Sigma-Aldrich Chemie GmbH, Schnelldorf, Germany)

Stannous (II) octoate (Sigma-Aldrich Chemie GmbH, Schnelldorf, Germany)

Toluene (Fisher Scientific GmbH, Schwerte, Germany)

Triethylamine (Sigma-Aldrich Chemie GmbH, Schnelldorf, Germany)

Valeraldehyde (Fisher Scientific GmbH, Schwerte, Germany)

Further Chemicals used for polymerisation reactions as, δ-valerolactone (VL, TCI, >98.0 %) and ε-caprolactone (CL, BLD pharma, 99.60 %) were dried overnight over CaH_2, then distilled. TBD (BLD pharma, 97 %), 1,8-Diazabicyclo[5.4.0]undec-7-ene (DBU, TCI, > 98.0 %), 1-[3,5-Bis(trifluoromethyl)phenyl]-3-cyclohexylthiourea (TU,TCI, > 98 %), polycaprolactone (Aldrich, Mn ~10,000 by GPC) were used as received. Benzyl alcohol (TCI, > 99 %) was degassed by repeated freeze-pump-thawing before storing under nitrogen.

Dichloromethane used in reactions (DCM, fluka, 99.8 %) was destilled, degassed and stored under nitrogen over a molecular sieve (4 Å).

Hydrogenations under high pressure were performed using Parr Series 5000 Multiple Reactor System (MRS) with 75 mL autoclaves. The system is designed for a maximum pressure of 200 bar. Before each reaction, the apparatus was flushed three times with nitrogen (20 – 30 bar), followed by three flushes with hydrogen (20 – 30 bar).

2 Analytics

For experiments requiring the exclusion of oxygen and moisture, the Schlenk technique was employed. Argon was used as inert gas. Also work on a Glovebox in nitrogen atmosphere was employed.

2.1 Nuclear Magnetic Resonance

NMR data was collected using a Bruker Avance III 500 or Avance III 500 HD at a frequency of 500 MHz (^{1}H NMR), 126 MHz (^{13}C NMR) or using a Bruker Avance Neo 400 spectrometer or 400 MHz (^{1}H NMR), 101 MHz (^{13}C NMR) in d-chloroform. The spectra were referenced to the residual solvent peak in ppm (d-chloroform: 7.26 ppm (s) for ^{1}H NMR and 77.16 ppm (t) for ^{13}C NMR) and analysed using Mestrenova. Chemical shifts are given in ppm within 0.1 ppm. The multiplicity of ^{1}H signals has been abbreviated as following: s = singulet, d = doublet, t = triplet, q = quadruplet, m = multiplet or combinations thereof. Coupling constants (J) are given in Hz within 0.1 Hz.

2.2 Gel permeation Chromatography

Molecular weights and molecular weight distributions (Đ) of the polymeric samples were determined using GPC. Data was collected using an Agilent 1260 Series Instrument with three Agilent SDV (8 mm x 300 mm) columns and an Agilent series G7162A RI detector. The method used had an eluent flow rate of 1mL/min, column temperature of 40 °C, sample concentration of 2 mg/mL, injection volume of 50 µl. Eluent has been chloroform and the system was calibrated against polystyrene(PS) standards (474-2 520 000 g/mol).

2.3 Differential scanning calorimetry & thermogravimetric analysis

Thermal analysis of the polymeric samples was performed by DSC under nitrogen atmosphere using a Netzsch DSC 204 F1 instrument between -100 and 200°C with a heating/cooling rate of 10 °C/min and TGA using a Netzsch STA 449 F5, between 30 and 590 °C with a heating rate of 10 °C/min in air atmosphere.

2.4 Structural analysis of lactones

Pentadecylcaprolactone (100 mg, 0.3 mmol, 1 eq.), tin(II) 2-ethylhexanoate (12.5 mg, 30 µmol, 10 mol%), and benzyl alcohol (333 mg, 3.1 mmol, 10 eq.) were combined in a 10 mL glass vial and heated to 130 °C. The reaction mixture was stirred for 2 h. Product composition was analysed by NMR spectroscopy.

3 Synthesis

3.1 General experimental protocols

3.1.1 Selective Hydrogenation of Cardanol (GP1)

An autoclave was charged with cardanol and Pd/C. The vessel was sealed and sequentially purged with nitrogen and hydrogen. Hydrogen pressure was then adjusted, and the reaction was carried out under controlled conditions (1000 rpm).

After cooling to room temperature, the heterogeneous catalyst was removed by syringe filtration (0.45 µm). The composition of the product mixture was determined by NMR spectroscopy. In most cases, the product mixture consisted of three compounds, a $C_{15}H_{31}$-substituted phenol (carda-phenol), a $C_{15}H_{31}$-substituted cyclohexanone (carda-ketone) and a $C_{15}H_{31}$-substituted cyclohexanol (carda-alcohol).

3.1.2 Ring-opening reaction of reaction mixture with succinic anhydride (GP2)

The crude hydrogenation product of cardanol was dissolved in acetonitrile, followed by the addition of succinic anhydride, triethylamine, and 4-dimethylaminopyridine. The reaction mixture was then refluxed. After completion, the solvent was removed under reduced pressure, and the residue was passed through a column of basic alumina using cyclohexane as the eluent. Evaporation of the eluent afforded the pure ketone.

3.1.3 Baeyer-Villiger oxidation under Mukaiyama conditions (GP3)

The substituted cyclohexanone (154 mg, 0.5 mmol, 1 eq.) and benzaldehyde (212 mg, 2 mmol, 4 eq.) were dissolved in 1,2-dichloroethane (4 mL) in a 10 mL glass vial. The vessel was flushed with oxygen and then sealed to maintain an oxygen atmosphere. The reaction mixture was stirred at 500 rpm for 24 h at 50 °C. Product composition was determined by NMR spectroscopic analysis.

3.1.4 Homo- and Copolymerisation of PDCL

All reactions have been carried out in nitrogen atmosphere either using Schlenk techniques or in a glovebox.

For an exemplary homopolymerisation, the catalyst (i.e. TBD, 4.3 mg, 0.03 mmol, 5 eq) and initiator BnOH (1.28 µl, 0.01 mmol, 2 eq) were solved in DCM (0.1 ml) and transferred to a Schlenk flask. The solvent was evaporated and PDCL (100 mg, 0.31 mmol, 50 eq) was added. The reaction was lwas achieved by cooling the reaction in liquid N_2. The product was purified by precipitation in cold methanol.

Copolymerisations of PDCL (i.e. 130 mg, 0.402 mmol, 50 Eq) with CL (i.e. 0.402 mmol, 50 Eq) have been carried out in bulk with the catalytic system of TBD (1.4 mg, 0.010 mmol, 1.25 Eq) and TU

(4.2 mg, 0.010 mmol, 1.25 Eq) on the initiator BnOH (1.04 µl, 0.010 mmol, 1.25 Eq). After stirring for a certain reaction time at 60°C NMR samples have been collected. The resulting polymer was precipitated in cold methanol.

For both, homo and copolymers, molar masses and the molar mass distribution were determined via GPC analysis ($CHCl_3$). The conversion was quantified by ^{1}H NMR, comparing the -CH_2-O- signals of the PDCL monomer (δ = 2.5-2.7 ppm) and the PDCL resulting polymer (δ = 2.3-2.2 ppm). ^{13}C NMR was used to analyse arrangement of PDCL and CL in the copolymers by doing dyad analysis. Thermal analysis using DSC and TGA have been completed.

3.2 Experimental protocols

3.2.1 Pressure screening of cardanol hydrogenation

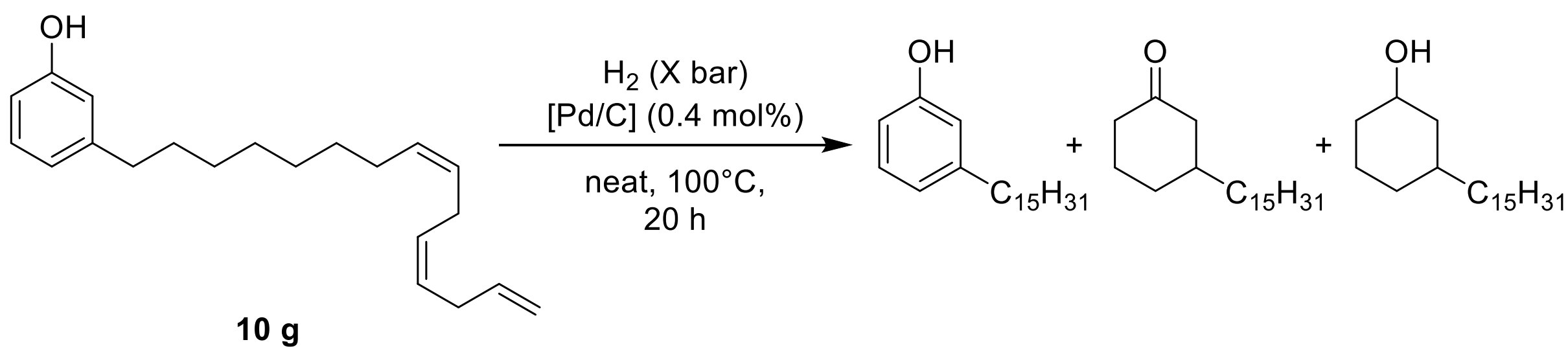

The hydrogenation was carried out according to GP 1 with different pressures of hydrogen, 100 °C and 20 h reaction time. The amounts used and calculated conversions are given in Table S1 and Table S2.

Table S1: Amounts used for hydrogenation of cardanol under different hydrogen pressures.

Run	p_{H2} / bar	m_{Phenol} / g	n_{Phenol} / mmol	$m_{Cat.}$ / mg	$n_{Cat.}$ / mmol	Loading / mol%
**1**	30	10.0	32.8	139	0.13	0.4
**2**	40	10.1	33.2	140	0.13	0.4
**3**	60	10.1	33.2	141	0.13	0.4
**4**	75	10.1	33.2	140	0.13	0.4

Table S2: Composition of product mixtures for hydrogenation of cardanol under different hydrogen pressures.

Run	Carda-Phenol / %	Carda-Alcohol / %	Carda-Ketone / %
**1**	25	0	75
**2**	7	10	83
**3**	9	12	79
**4**	7	29	64

3.2.2 Kinetics of cardanol hydrogenation

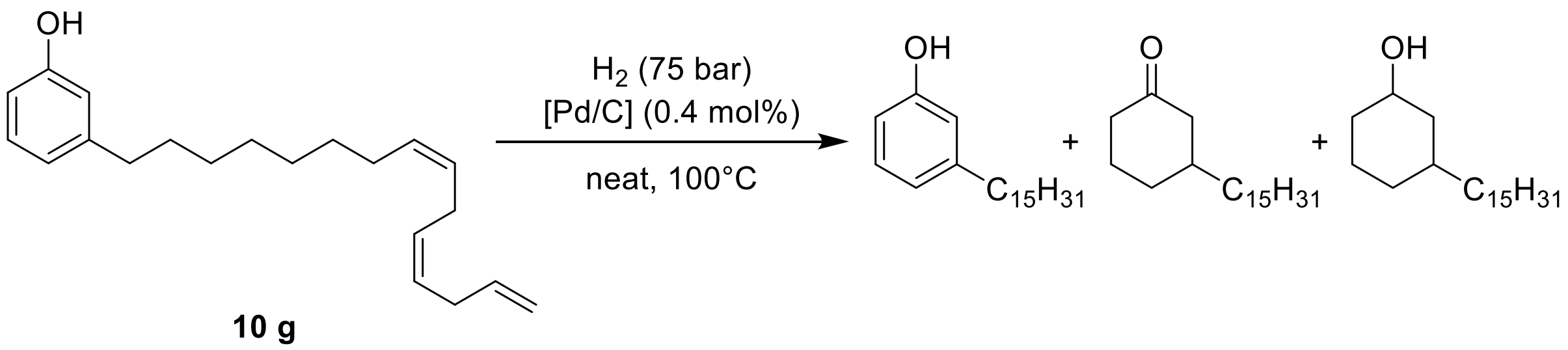

The hydrogenation was carried out according to GP 1 with different reaction times, 100 °C and 75 bar of hydrogen. The amounts used and calculated conversions are given in Table S3 and Table S4.

Table S3: Amounts used for kinetics of cardanol hydrogenation.

Run	*t* / h	m_{Phenol} / g	n_{Phenol} / mmol	$m_{Cat.}$ / mg	$n_{Cat.}$ / mmol	Loading / mol%
**1**	2	10.0	32.8	141	0.13	0.4
**2**	4	10.0	32.8	141	0.13	0.4
**3**	20	10.0	32.8	141	0.13	0.4
**4**	24	10.1	33.2	140	0.13	0.4

Table S4: Composition of product mixtures for kinetics of cardanol hydrogenation.

Run	Carda-Phenol / %	Carda-Alcohol / %	Carda-Ketone / %
**1**	39	5	56
**2**	28	5	67
**3**	20	8	72
**4**	0	32	68

3.2.3 Purification of ketone

3.2.3.1 Column chromatography

For small scale reactions, the crude product was purified via automated column chromatography (NP: Biotage® SNAP-Ultra 50 g cyclohexane: ethyl acetate, 3 CV 4% EtOAc, 1 CV 4% EtOAc, 9.1 CV 4-30% EtOAc, 100 mL/min; RP: Biotage® Sfär C18 Duo 100 Å 30 µm acetonitrile, acetic acid (0.1%), 50 mL/min).

3.2.3.2 Ring-opening reaction of succinic anhydride with carda-phenol

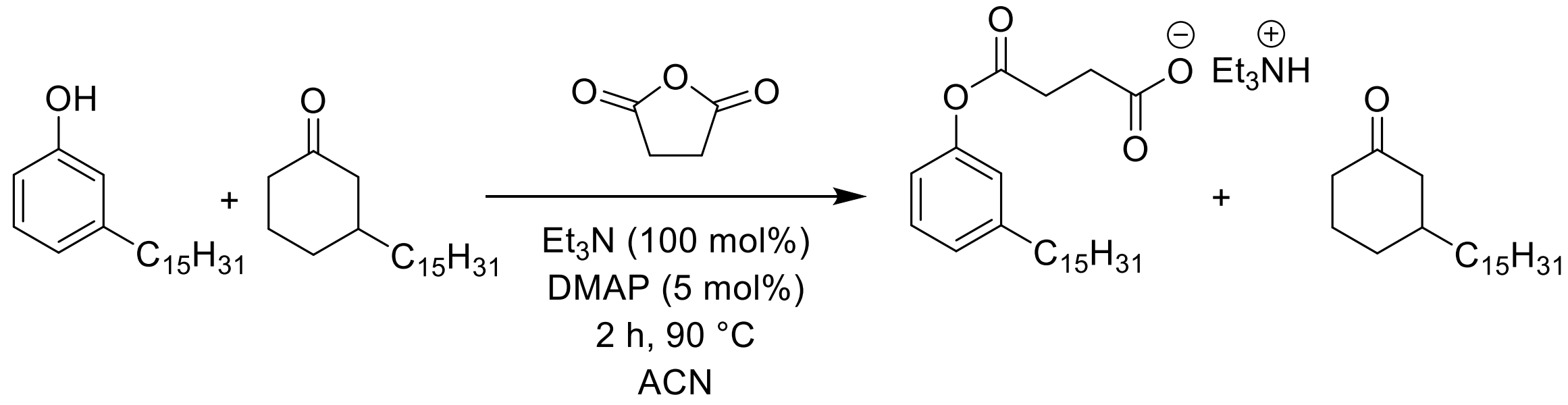

The ring-opening reaction of succinic anhydride with crude product of cardanol hydrogenation was carried out according to GP 2. For this, a mixture of carda-phenol (48%) and carda-ketone (52%) was used and converted with different amounts of succinic anhydride. The equivalents of anhydride are always in relation to the total amount of carda-phenol and carda-alcohol combined. The amounts used and calculated conversions are given in Table S5 and Table S6.

Table S5: Amounts used for ring-opening reaction of succinic anhydride with alcoholic species in crude product of cardanol hydrogenation.

Run	$m_{Substrates}$ / g	$n_{Substrates}$ / mmol	$m_{Anhydride}$ / g	$n_{Anhydride}$ / mmol	Eq. Anhydride
**1**	1.53	5.02	0.49	4.9	1
**2**	1.51	4.96	0.98	9.8	2
**3**	1.52	4.98	1.48	14.8	3

Table S6: Composition of product mixtures for ring-opening reaction of succinic anhydride with alcoholic species in crude product of cardanol hydrogenation.

Run	Res. carda-phenol / %	Succ. carda-phenol / %	carda-ketone / %
**1**	31	19	50
**2**	18	31	51
**3**	8	40	52

3.2.3.3 Purification using triethylamine

Purification of the resulting ketone was achieved by dissolving the crude product in cyclohexane (approximately 250 g/L) and adding triethylamine (100 wt%). The solution was filtered through a column of basic alumina (4 cm diameter, 30 cm height), after which the solvent and residual triethylamine were removed by vacuum distillation. This procedure afforded the ketone in 85% yield and >99% purity.

3.2.4 Baeyer-Villiger oxidation of carda-ketone with *m*CPBA

Carda-ketone (10 g, 32.4 mmol, 1 eq.) was dissolved in dichloromethane (40 mL). *m*CPBA (75%, 11.2 g, 48.6 mmol, 1.5 eq.) were added portion wise over 30 min. The reaction mixture was allowed to react over 90 min at room temperature. Any left-over peracid and formed carboxylic acid was washed out with NaOH solution (10%, 3 x 30 mL) and brine (1 x 30 mL) and the organic phase was dried over $MgSO_4$. After filtering off the drying agent and removing the solvent *in vacuo*, the lactones were obtained in a yield of 84% as a colourless solid.

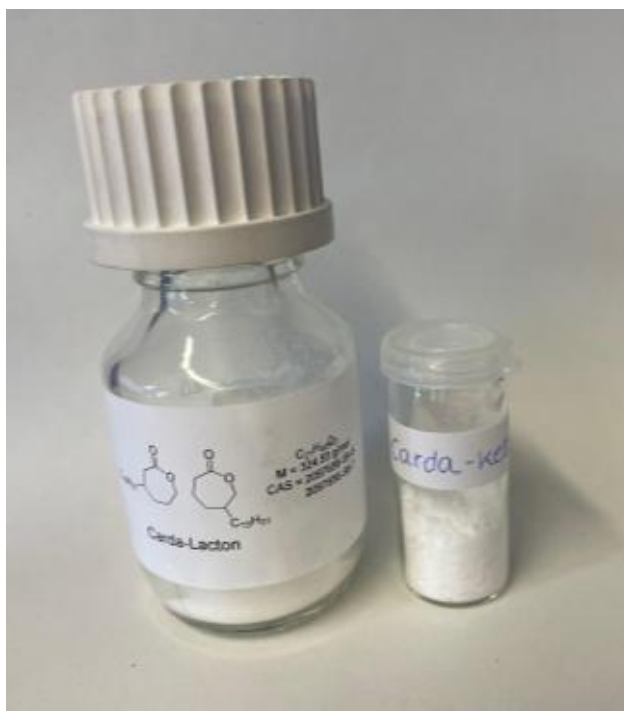

Figure S1: Lactone as end product on the left and ketone on the right.

3.2.5 Baeyer-Villiger oxidation of carda-ketone under Mukaiyama conditions

3.2.5.1 BVO of carda-ketone with different amounts of benzaldehyde

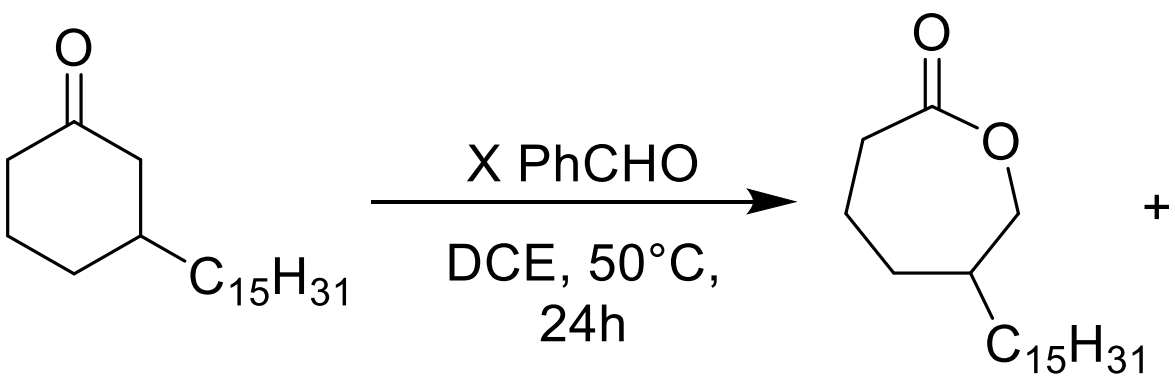

BVO of carda-ketone was carried out according to GP 3 with different equivalents of benzaldehyde. Product composition was determined by NMR spectroscopic analysis (Table S7).

Table S7: BVO of carda-ketone with different amounts of benzaldehyde.

Benzaldehyde equivalents	Conversion to lactones / %
**1**	9
**1.5**	26
**2**	49
**3**	75
**5**	>99
**10**	>99

3.2.5.2 BVO of carda-ketone in different solvents

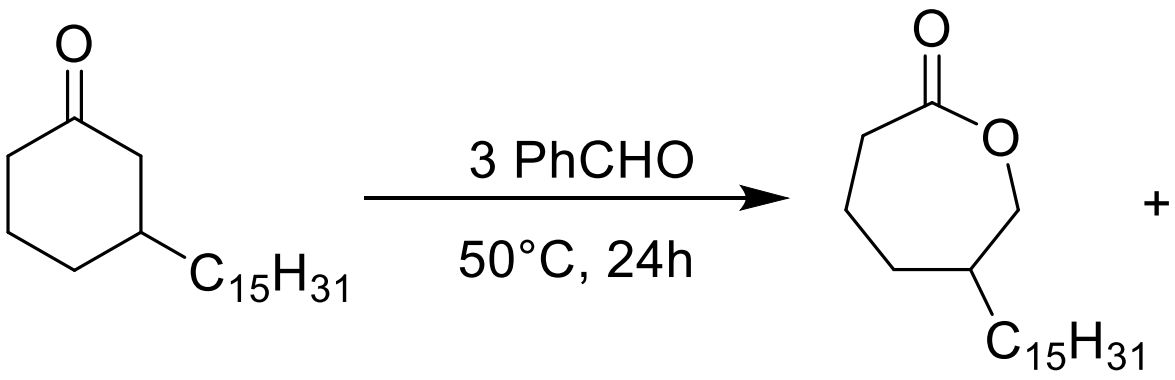

BVO of carda-ketone was carried out according to GP 3 with different solvents. Product composition was determined by NMR spectroscopic analysis (Table S8).

Table S8: BVO of carda-ketone in different solvents.

Solvent	Conversion to lactones / %
**Acetonitrile**	0
**Ethanol**	0
**Cyclohexane**	30
**1,2-Dichloroethane**	44
**Ethyl acetate**	37

3.2.5.3 BVO of carda-ketone with different aldehydes as cosubstrates

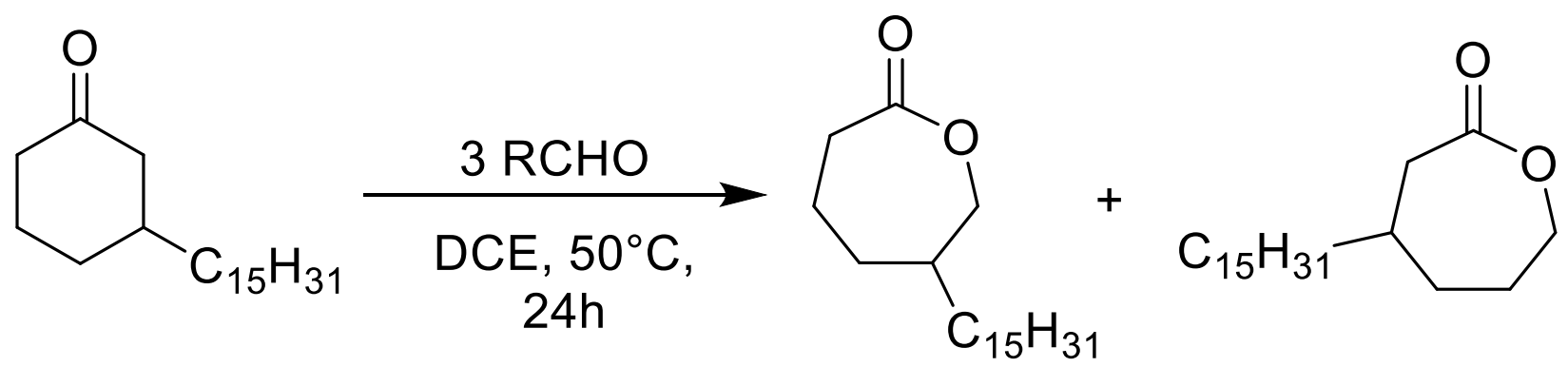

BVO of carda-ketone was carried out according to GP 3 with different aldehydes. Product composition was determined by NMR spectroscopic analysis (Table S9Table S9).

Table S9: BVO of carda-ketone using different aldehydes as cosubstrate.

Solvent	Conversion to lactones / %
**Benzaldehyde**	86
**Butyraldehyde**	47
**Isobutyraldehyde**	27
**Valeraldehyde**	39
**Heptanal**	26

3.2.6 Polymerisation of PDCL

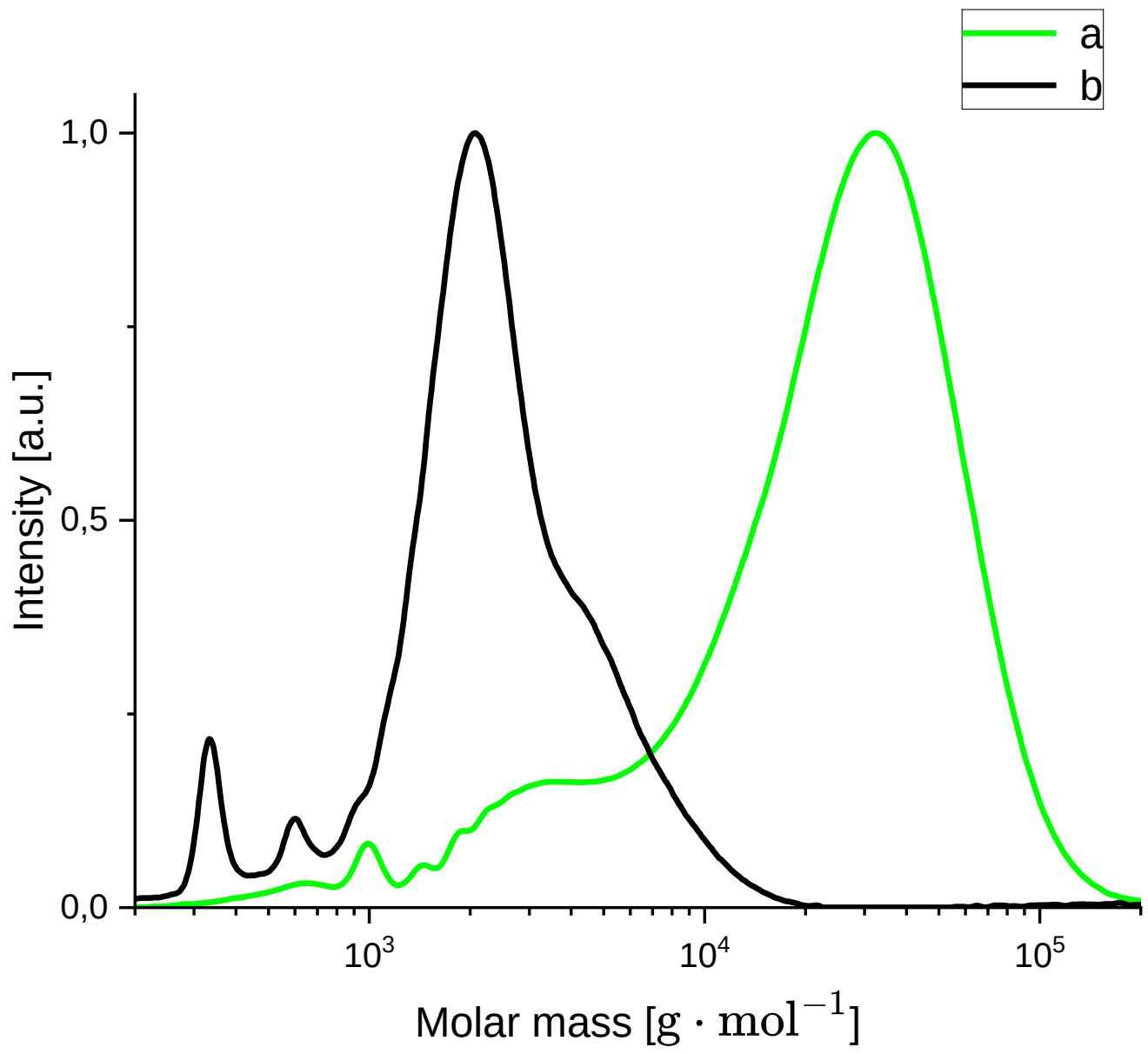

Figure S2: GPC traces of polymers obtained under varying conditions.

Conditions: a: TBD-catalysed, monomer/catalyst/initiator = 200:2:5 (molar), bulk, 60 °C, 94 % conversion (^{1}H NMR)

b: DBU-catalysed, monomer/catalyst/initiator = 50:2:5 (molar), bulk, 110 °C, 71% conversion (^{1}H NMR)

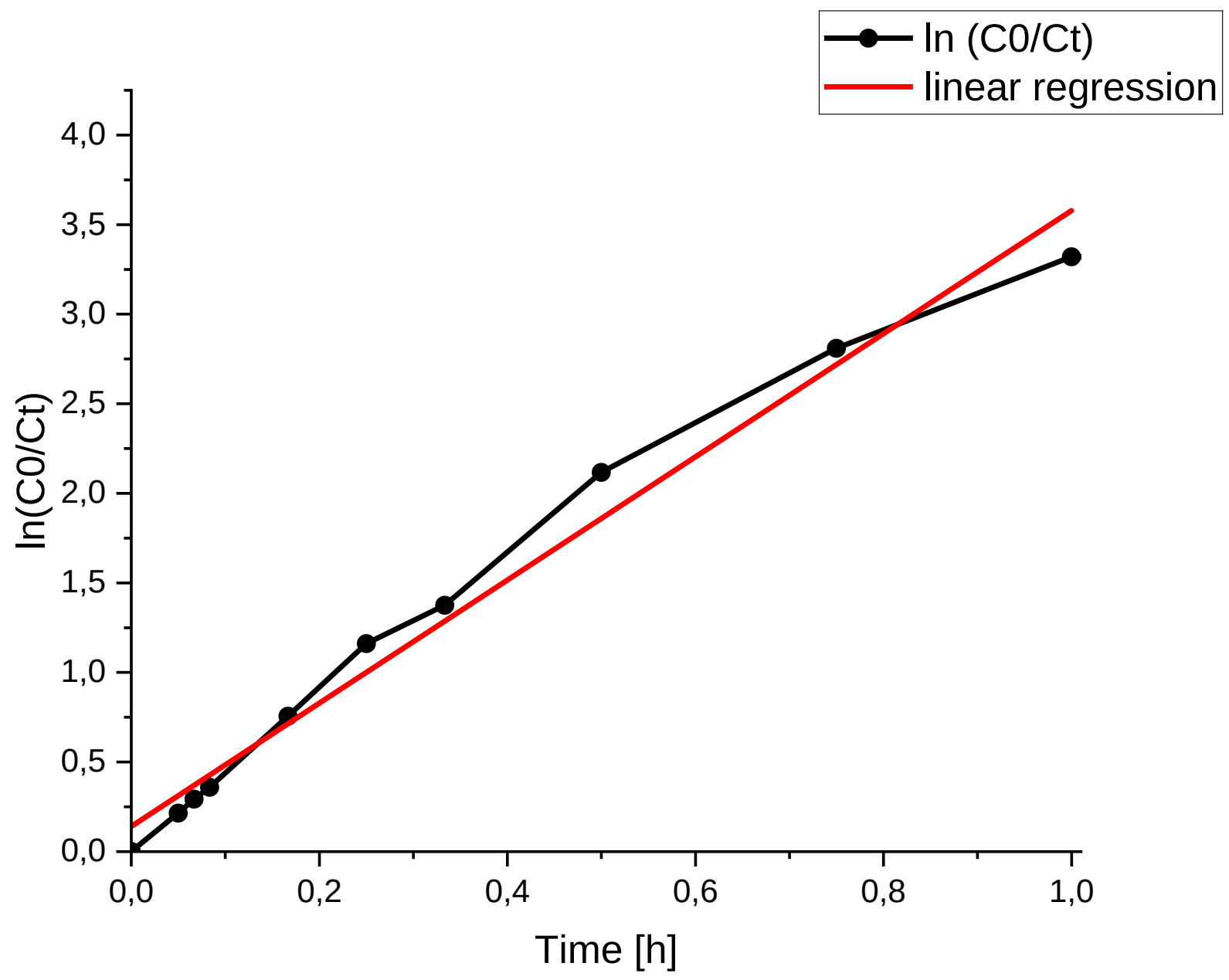

Figure S3: $\boldsymbol{ln\left(\frac{C_0}{C_t}\right)}$ **vs. time for polymerisation at 60 °C.**

Conditions: TBD-catalysed, monomer/catalyst/initiator = 50:2:5 (molar), bulk.

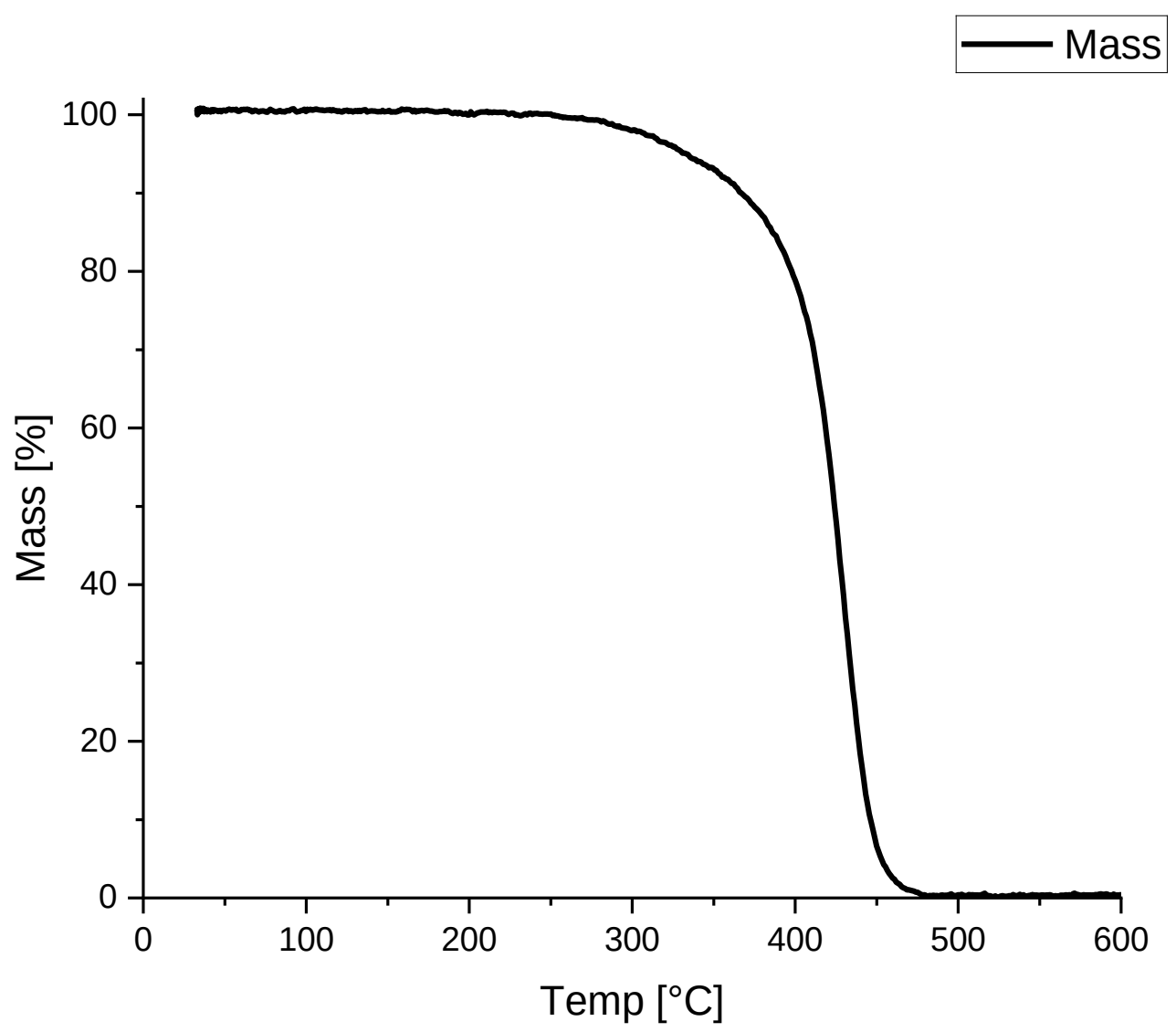

Figure S4: TGA measurements for poly(PDCL) resulting from

PDCL/TBD/BnOH = 1000:1:100 (molar), bulk.

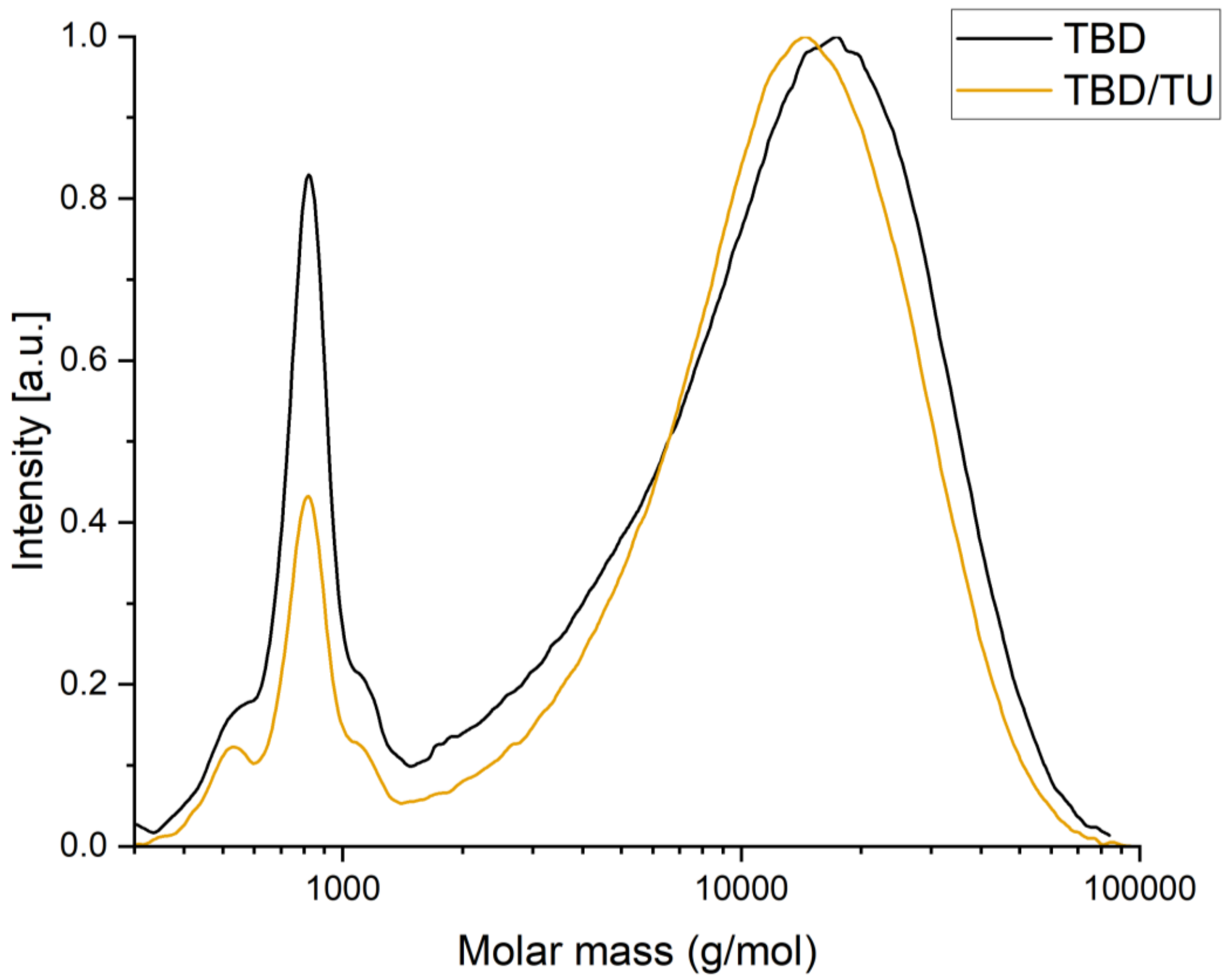

Figure S5: GPC trace of copolymer of 50:50 PDCL:CL. a: catalysed with 2.5 mol % TBD. b: catalysed with 2.5 mol % TBD and 2.5 mol % TU. The low molecular weight peak (<1000 g/mol) results from monomeric and oligomeric PDCL residues within the samples.

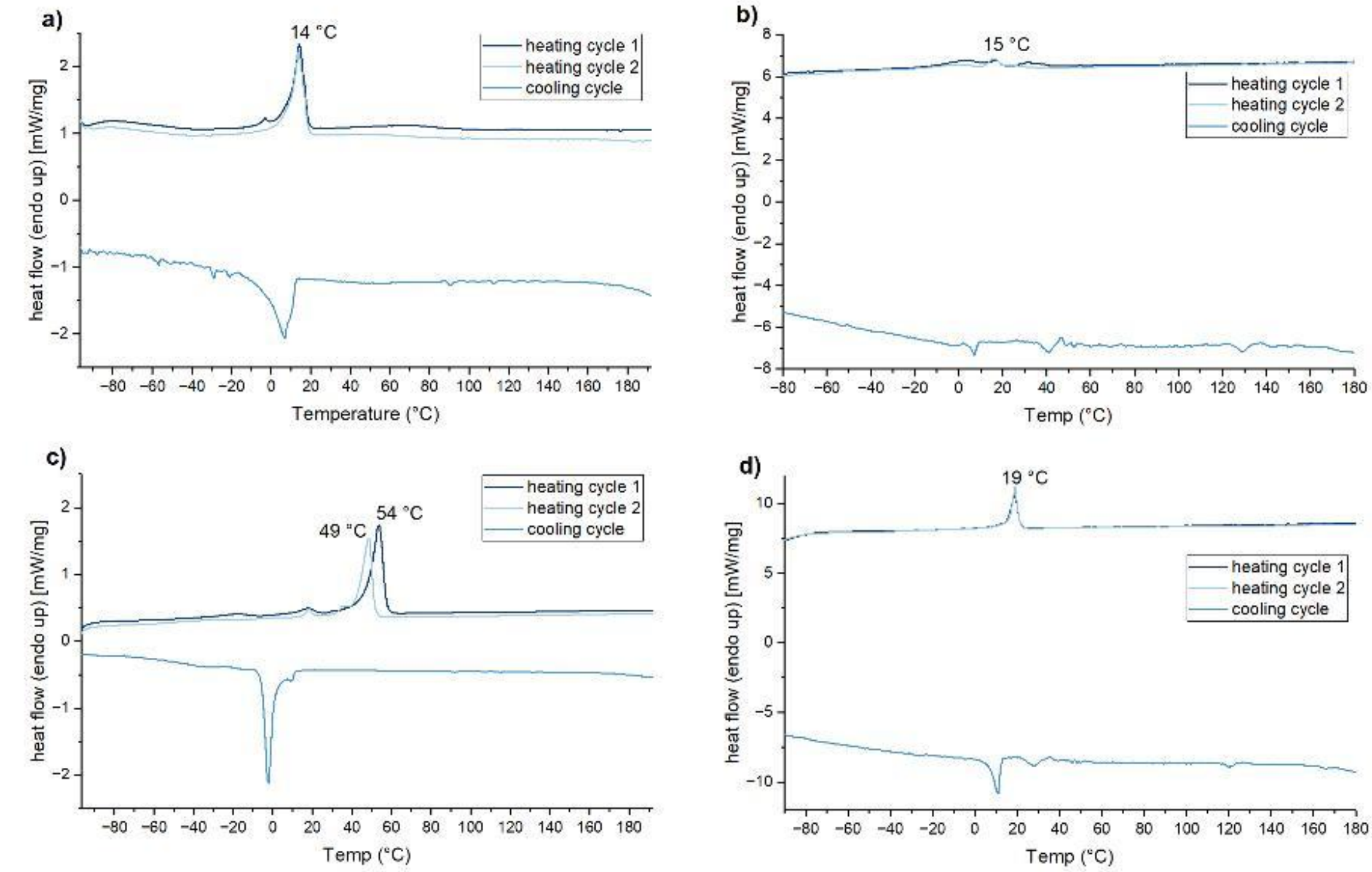

Figure S6: DSC thermograms of copolymer PDCL:CL of varying ratios. Heating rate = 10 K min^{-1}, from 100-200 °C. Copolyester resulting from a) PDCL:CL 50:50. b) PDCL:CL 30:70. c) PDCL:CL 10:90. d) PDCL:CL 70:30.

4 NMR-Spectra

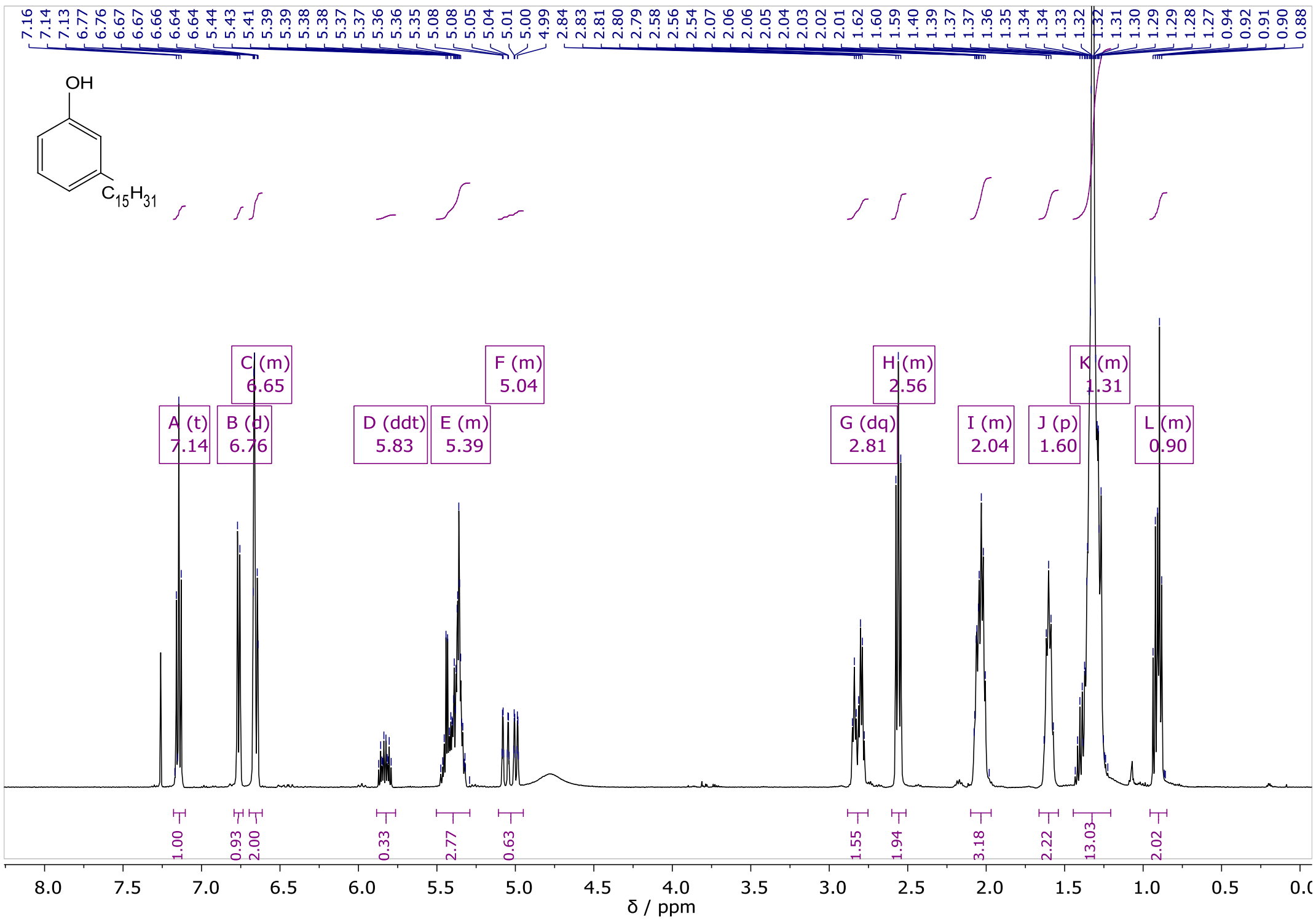

Figure S7: ^{1}H-NMR ($CDCl_3$, 500 MHz, 298K) spectrum of cardanol.

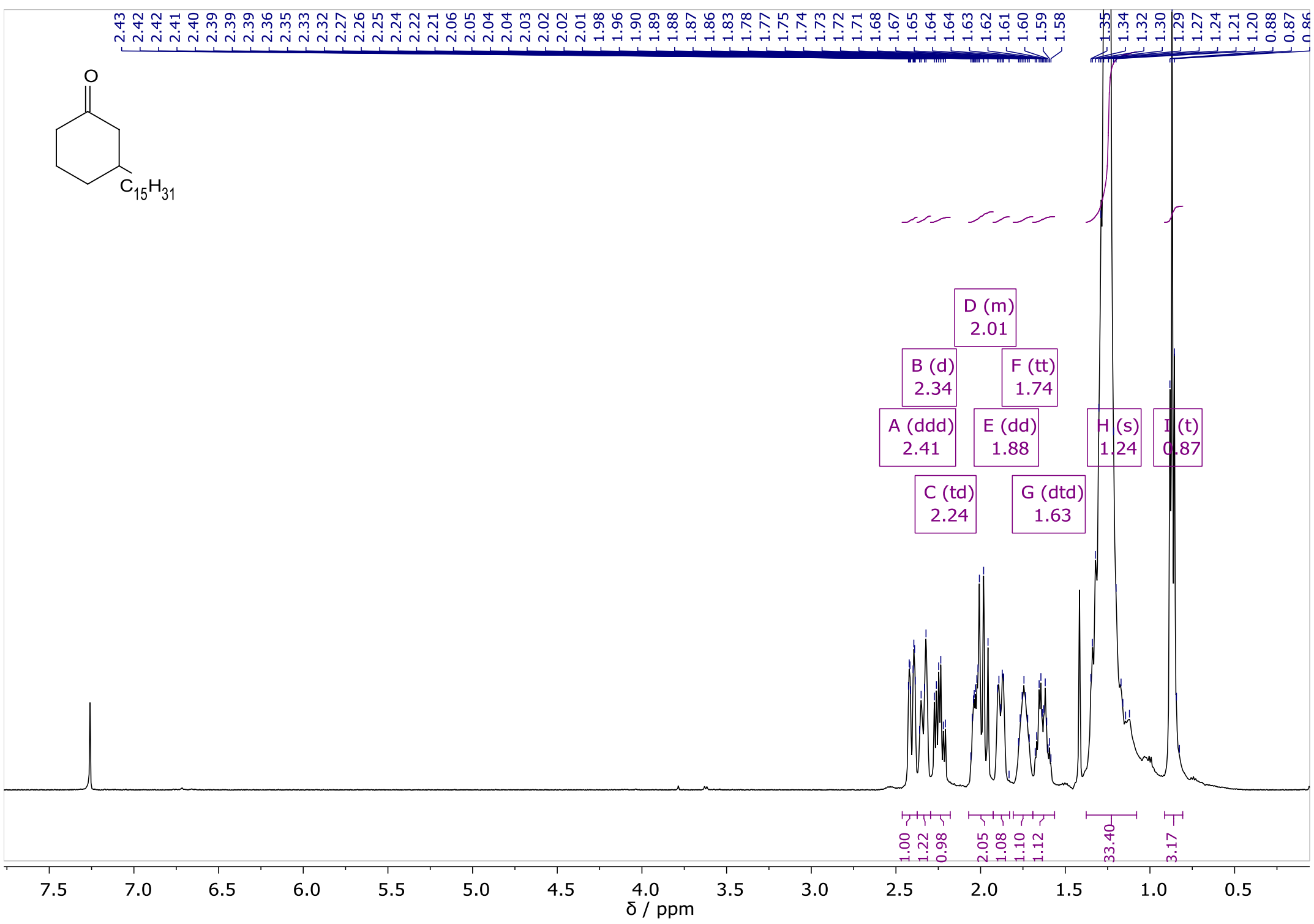

Figure S8: ^{1}H-NMR ($CDCl_3$, 500 MHz, 298K) spectrum of carda-ketone.

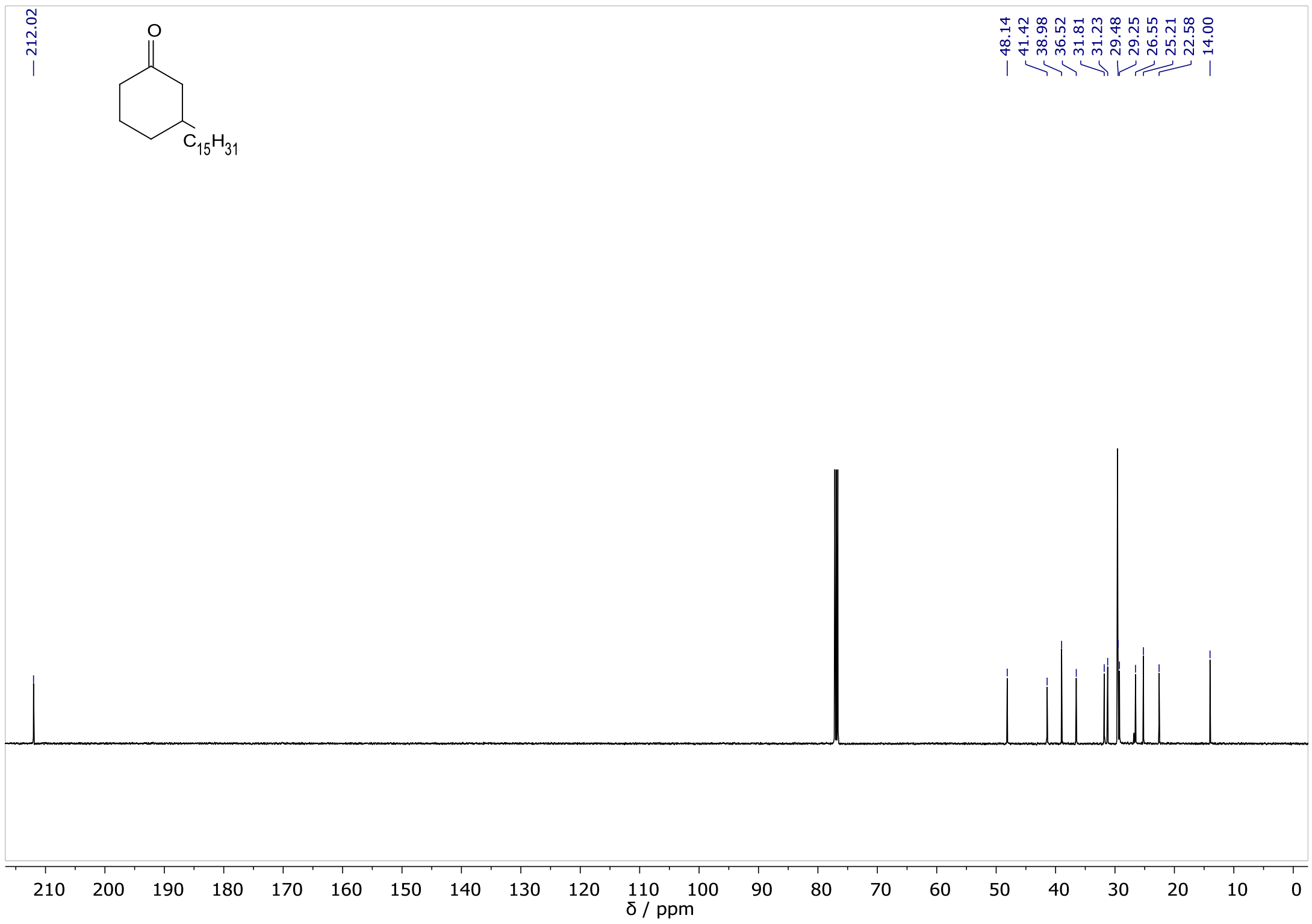

Figure S9: ^{13}C-NMR ($CDCl_3$, 125 MHz, 298K) spectrum of carda-ketone.

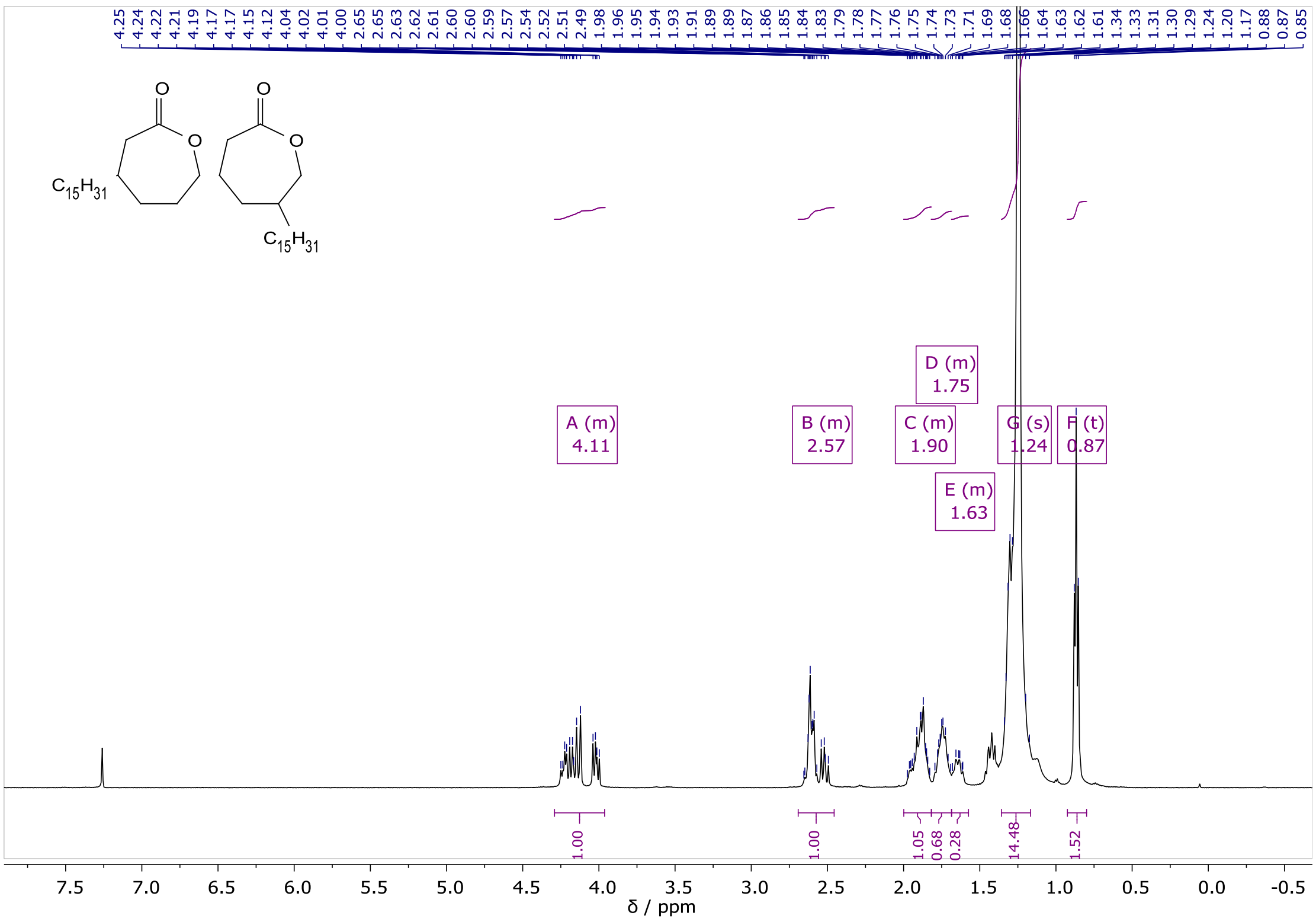

Figure S10: ^{1}H-NMR ($CDCl_3$, 500 MHz, 298K) spectrum of PDCL.

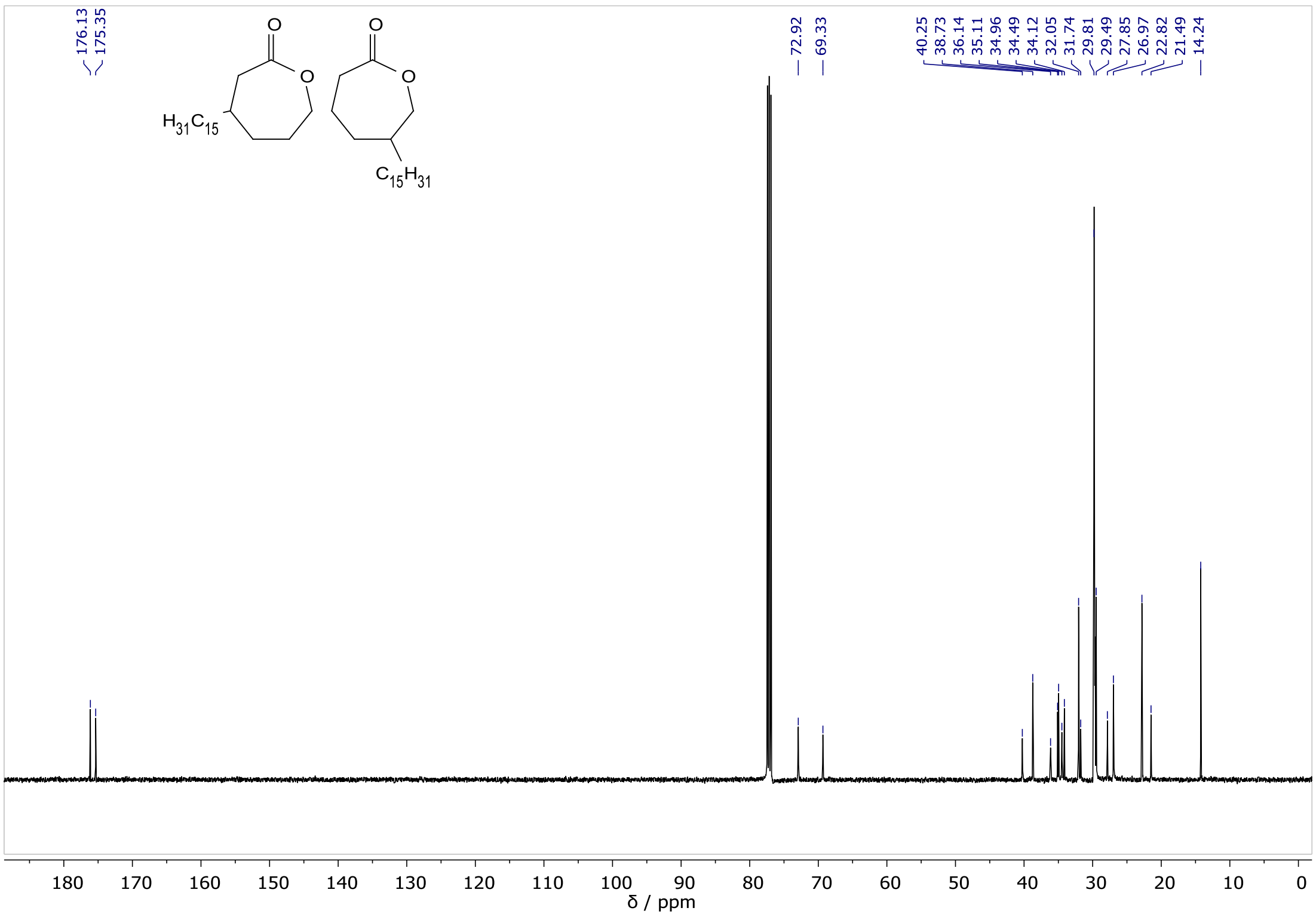

Figure S11: ^{13}C-NMR ($CDCl_3$, 125 MHz, 298K) spectrum of PDCL.

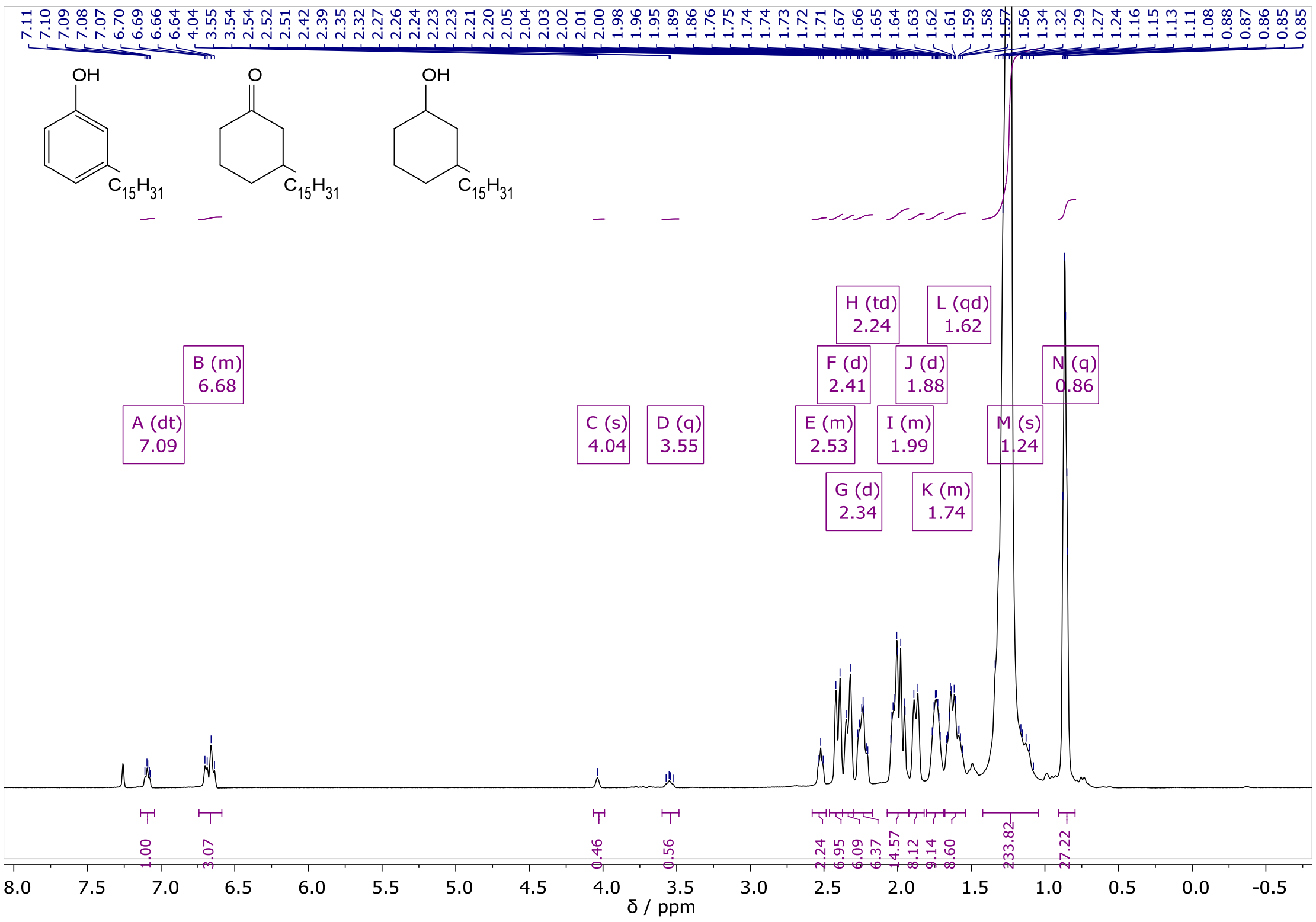

Figure S12: ^{1}H-NMR ($CDCl_3$, 500 MHz, 298K) spectrum of cardanol hydrogenation crude product.

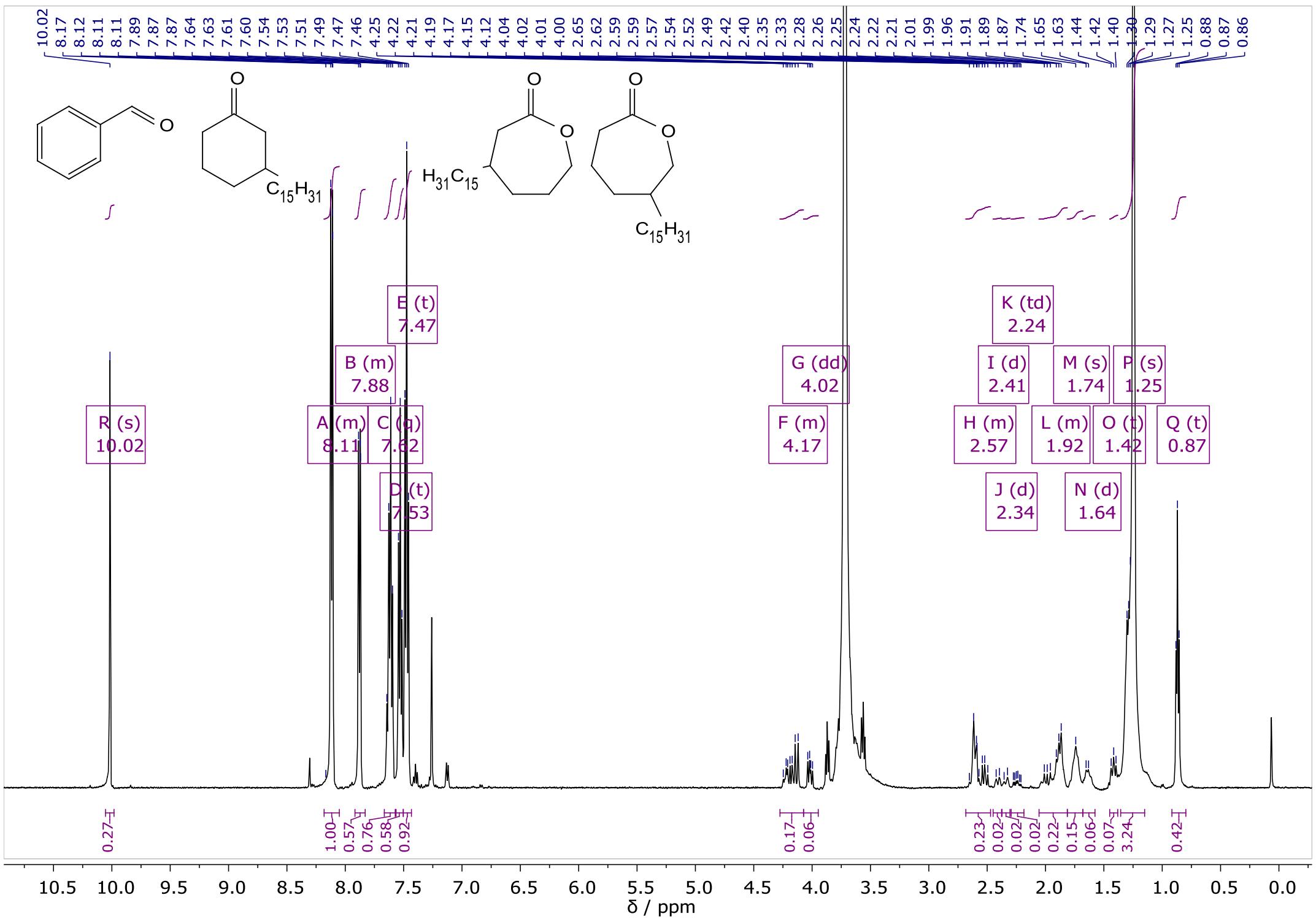

Figure S13: ^{1}H-NMR ($CDCl_3$, 500 MHz, 298K) spectrum of BVO of carda-ketone with benzaldehyde.

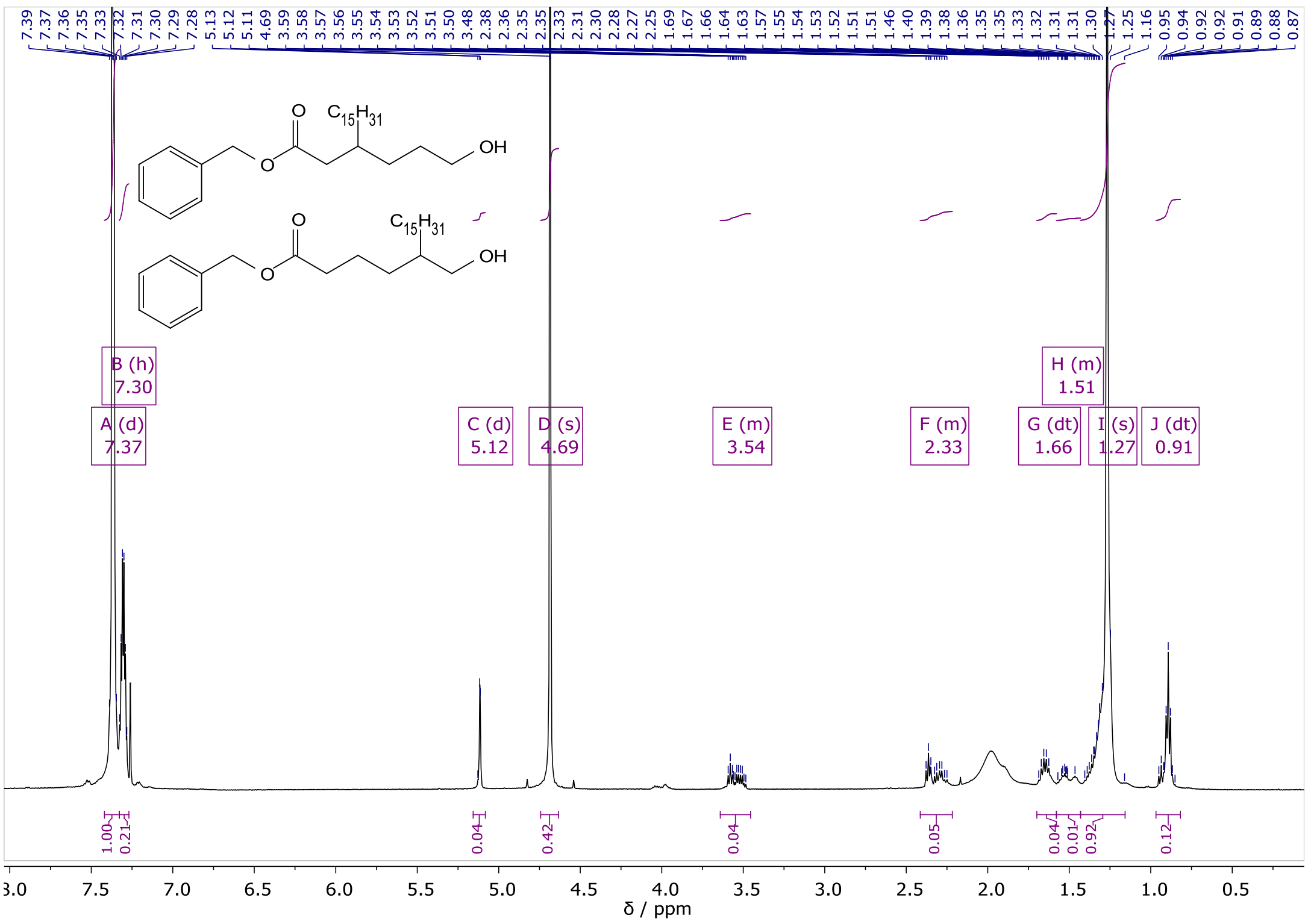

Figure S14: ^{1}H-NMR ($CDCl_3$, 500 MHz, 298K) spectrum of ring-opening reaction of PDCL with benzyl alcohol.

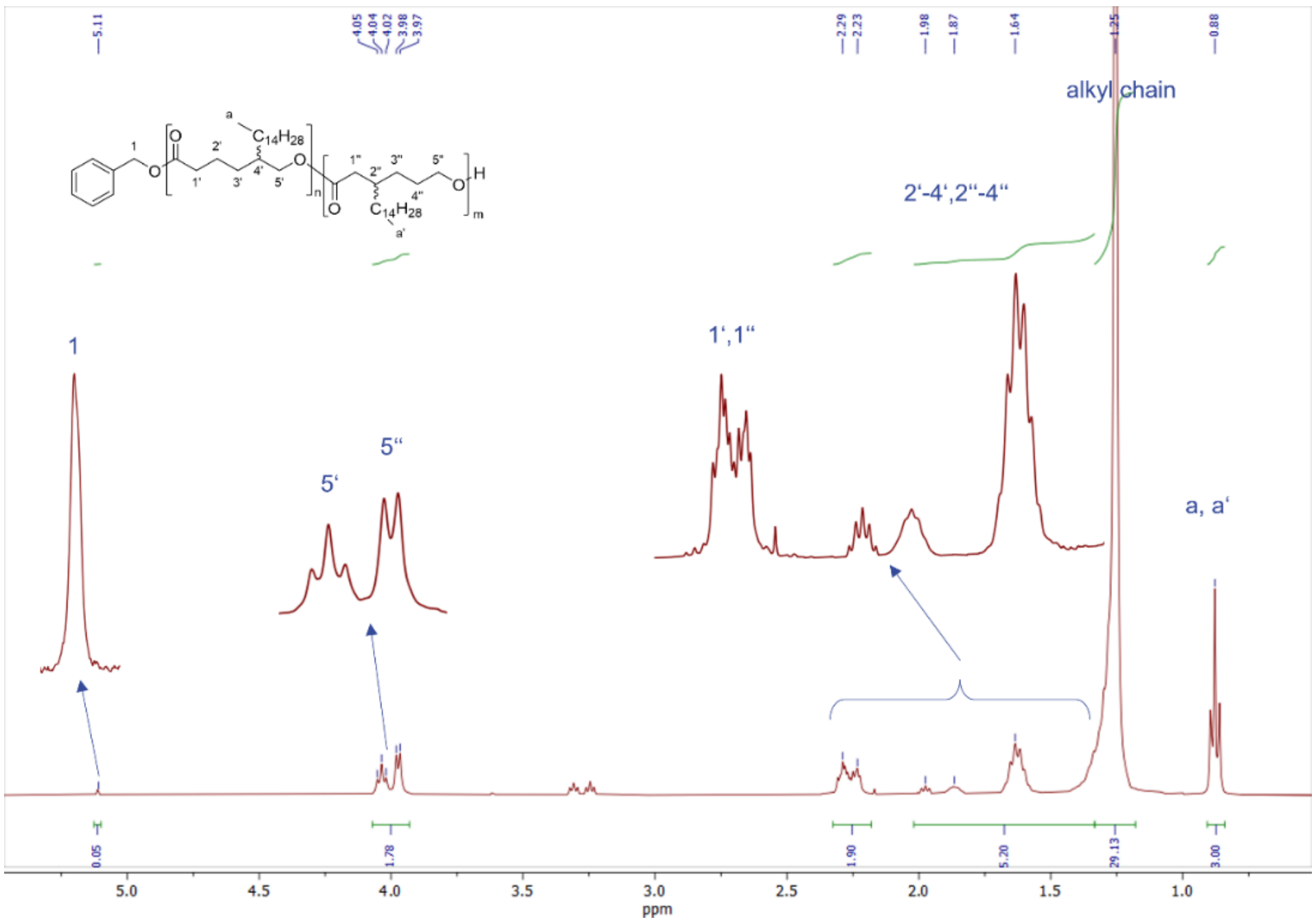

Figure S15: ^{1}H NMR ($CDCl_3$, 400 MHz, 298K) spectrum of homopolymerisation of PDCL.

Conditions: TBD-catalysed, monomer/catalyst/initiator = 50:2:5 (molar), bulk, 60 °C.

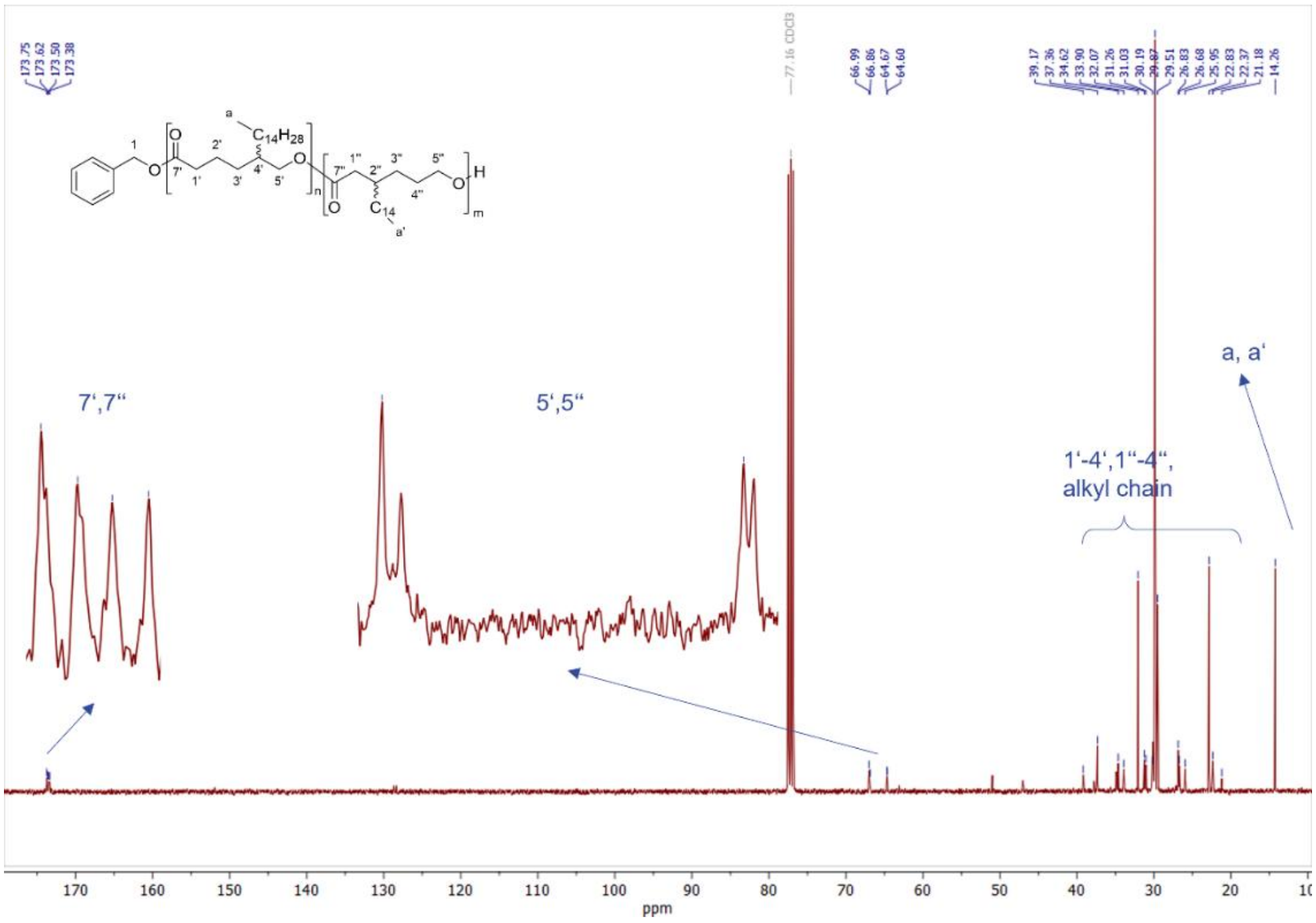

Figure S16: ^{13}C NMR ($CDCl_3$, 100 MHz, 298K) spectrum of poly(PDCL) in $CDCl_3$

Conditions: TBD-catalysed, monomer/catalyst/initiator = 50:2:5 (molar), bulk, 60 °C.

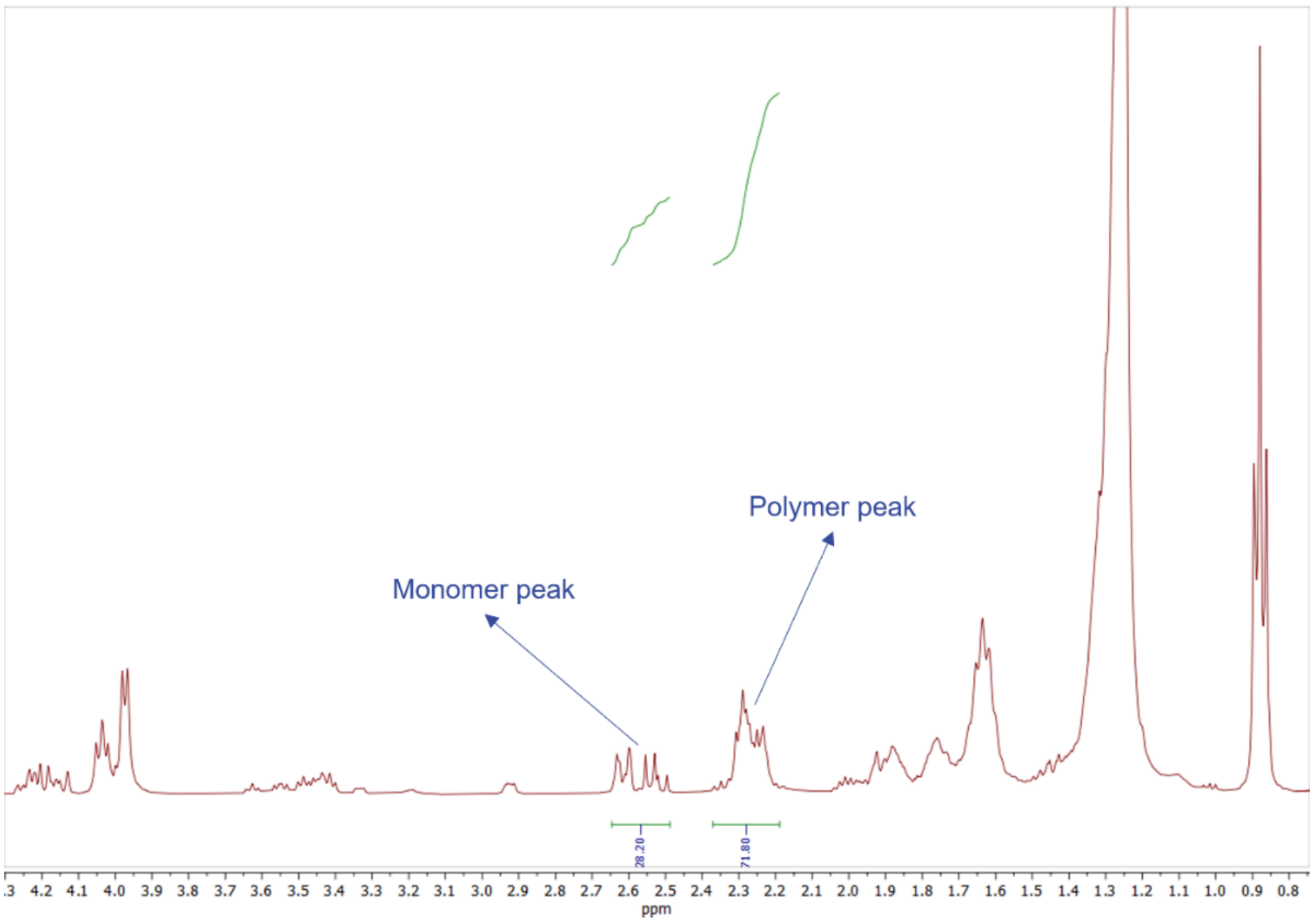

Figure S17: Exemplary quantification of polymer conversion in a ^{1}H NMR ($CDCl_3$, 400 MHz, 298K) spectrum.

Conditions: DBU-catalysed, monomer/catalyst/initiator = 50:2:5 (molar), bulk, 110 °C.

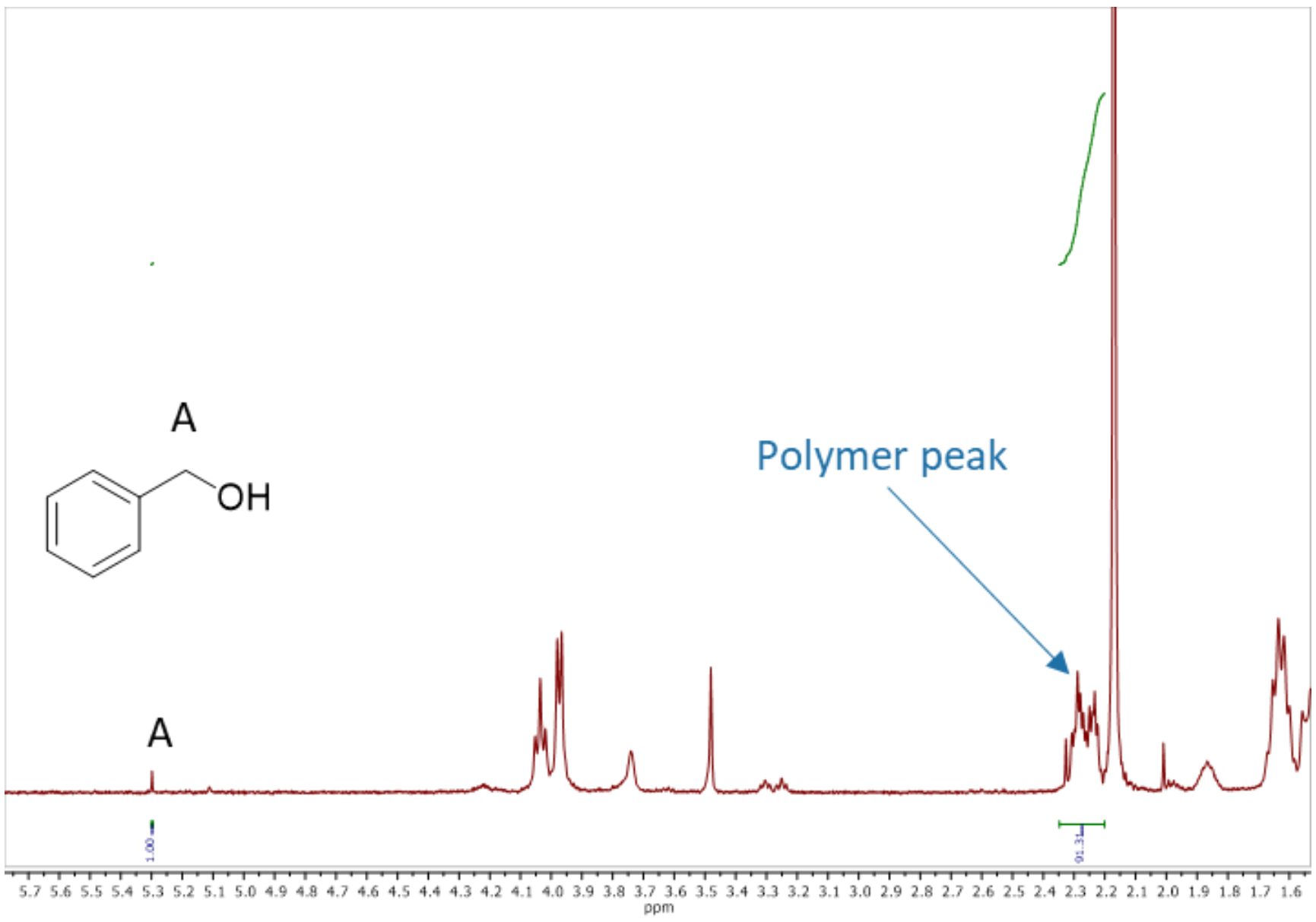

Figure S18: Exemplary quantification of polymer repeating units in a ^{1}H NMR ($CDCl_3$, 400 MHz, 298K) spectrum.

Conditions: TBD-catalysed, monomer/catalyst/initiator = 200:2:5 (molar), bulk, 60 °C.

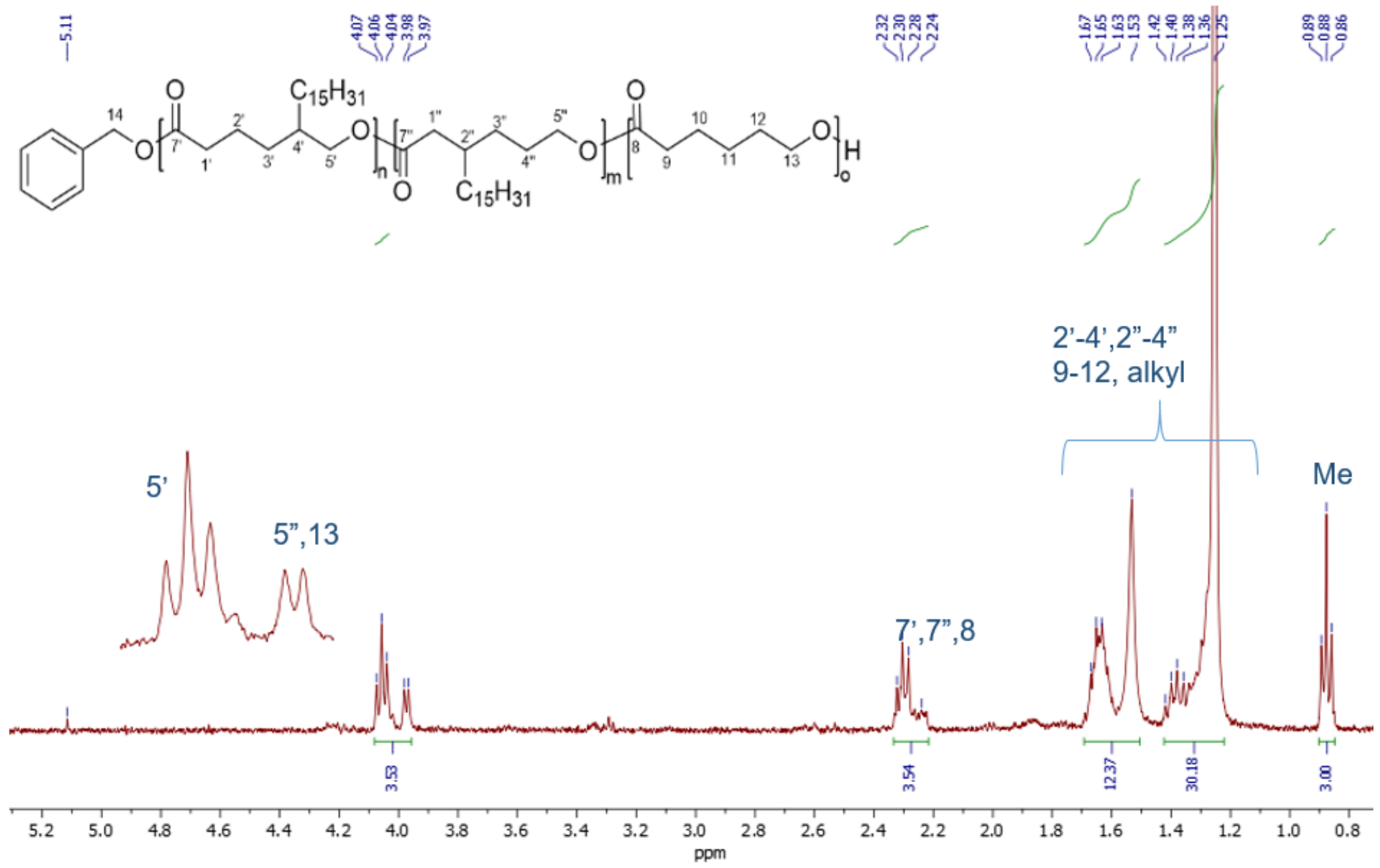

Figure S19: ^{1}H NMR ($CDCl_3$, 400 MHz, 298K) spectrum of copolymer: PDCL – ε-caprolactone in $CDCl_3$.

Conditions: TBD/TU-catalysed, PDCL/CL/catalyst/initiator = 50:50:1.25:1.25 (molar), bulk, 60 °C.

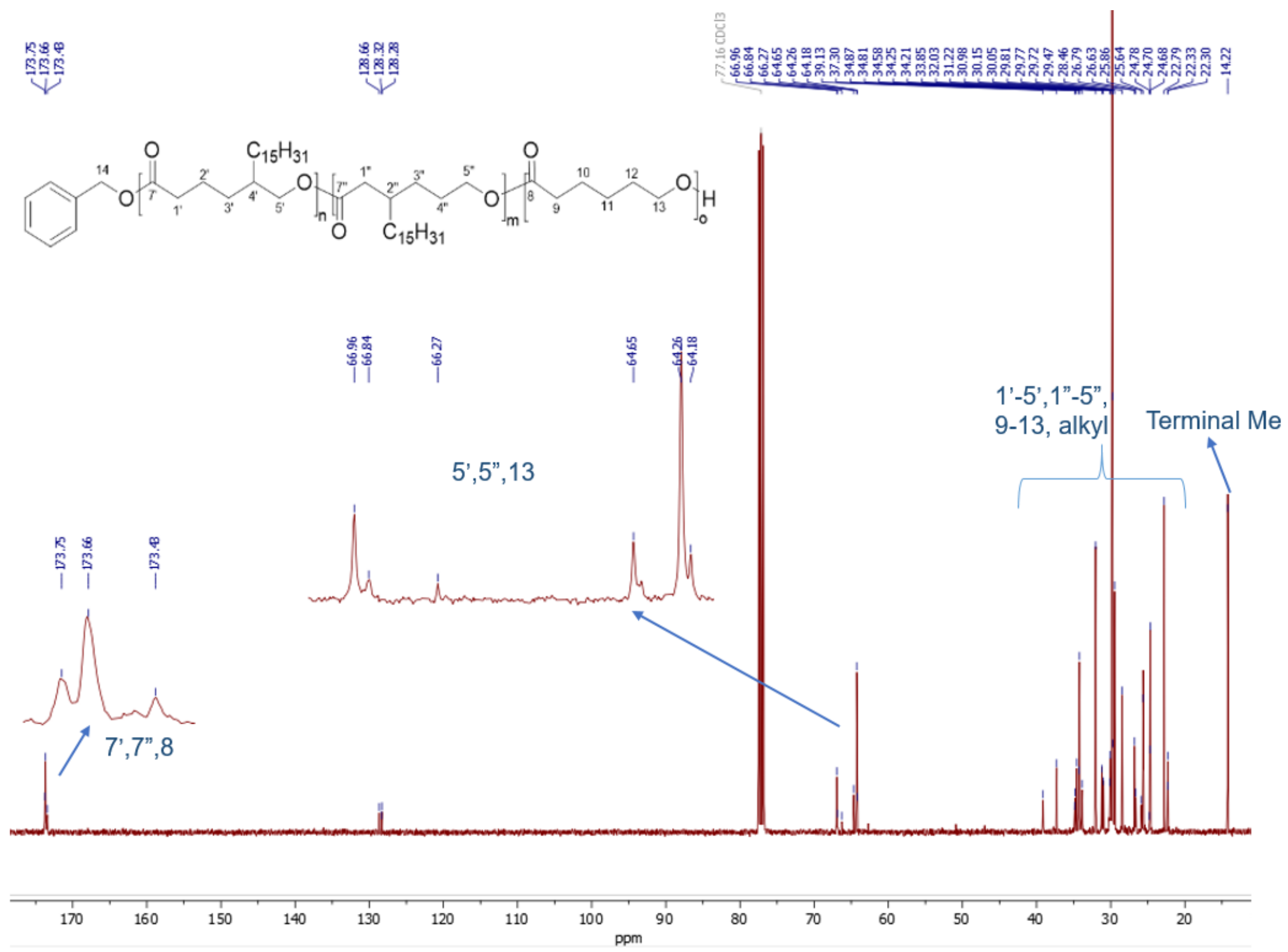

Figure S20: ^{13}C NMR ($CDCl_3$, 100 MHz, 298K) spectrum of copolymer: PDCL – ε-caprolactone in $CDCl_3$.

Conditions: TBD/TU-catalysed, PDCL/CL/catalyst/initiator = 50:50:1.25:1.25 (molar), bulk, 60 °C.

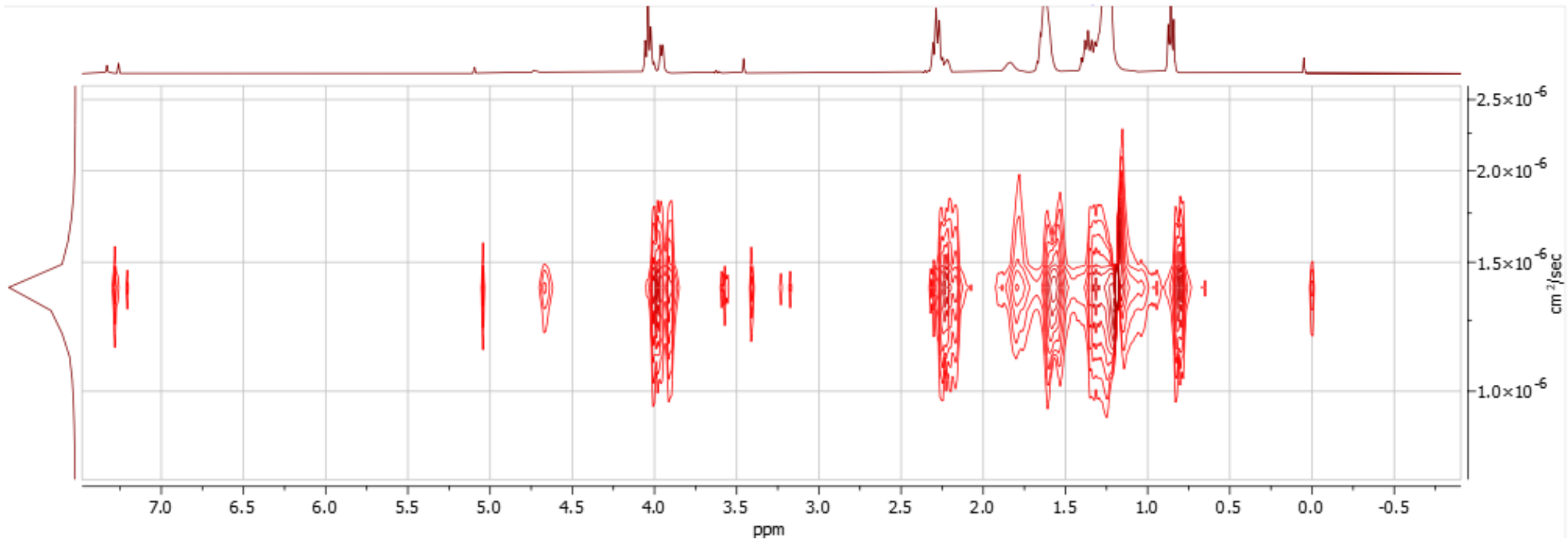

Figure S21: DOSY($CDCl_3$, 400 MHz, 298K) spectrum of copolymer 50:50 PDCL:CL.

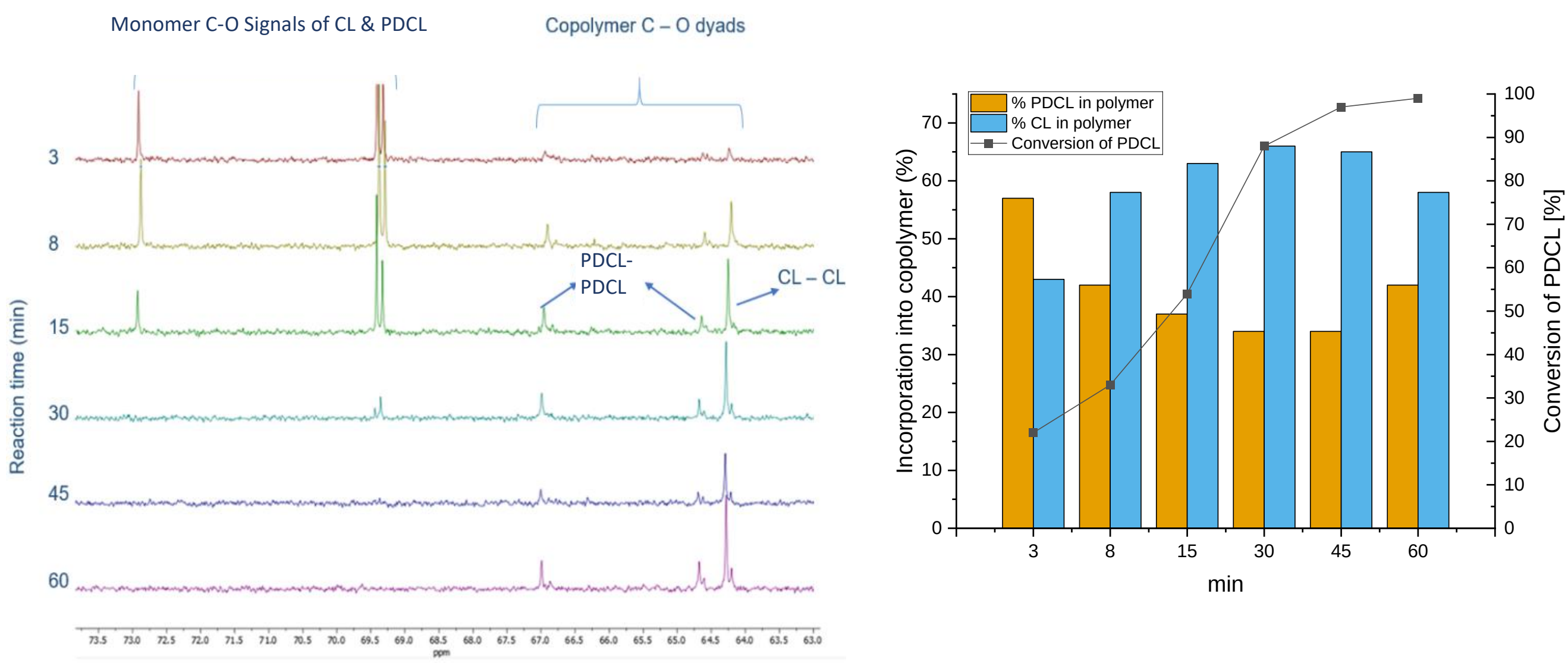

Figure S22: left:^{13}C NMR ($CDCl_3$, 100 MHz, 298K) spectra after certain time intervals of copolymerisation of PDCL – CL in $CDCl_3$. C-O Signals of the Monomers (73ppm CL, 69,5ppm PDCL isomers) are observed downfield, copolymer homodyads of CL-CL and PDCL-PDCL are shifted upfield. Right: percentual incorporation of monomers PDCL and CL into the polymer over time. Overlaid is a line showing the conversion of PDCL over time, derived from ^{1}H NMR

Conditions: TBD/TU-catalysed, PDCL/CL/catalyst/initiator = 50:50:1.25:1.25 (molar), bulk, 60 °C.
